# Association of Prenatal Exposure to Endocrine-Disrupting Chemicals With Liver Injury in Children

**DOI:** 10.1001/jamanetworkopen.2022.20176

**Published:** 2022-07-06

**Authors:** Vishal Midya, Elena Colicino, David V. Conti, Kiros Berhane, Erika Garcia, Nikos Stratakis, Sandra Andrusaityte, Xavier Basagaña, Maribel Casas, Serena Fossati, Regina Gražulevičienė, Line Småstuen Haug, Barbara Heude, Léa Maitre, Rosemary McEachan, Eleni Papadopoulou, Theano Roumeliotaki, Claire Philippat, Cathrine Thomsen, Jose Urquiza, Marina Vafeiadi, Nerea Varo, Miriam B. Vos, John Wright, Rob McConnell, Martine Vrijheid, Lida Chatzi, Damaskini Valvi

**Affiliations:** 1 Department of Environmental Medicine and Public Health, Icahn School of Medicine at Mount Sinai, New York City, New York; 2 Department of Population and Public Health Sciences, Keck School of Medicine, University of Southern California, Los Angeles; 3 Department of Biostatistics, Columbia University, New York City, New York; 4 Department of Environmental Sciences, Vytautas Magnus University, Kaunas, Lithuania; 5 ISGlobal, Barcelona, Spain; 6 Universitat Pompeu Fabra, Barcelona, Spain; 7 Centro de Investigación Biomédica en Red Epidemiología y Salud Pública, Madrid, Spain; 8 Norwegian Institute of Public Health, Oslo, Norway; 9 Université de Paris Cité, Institut National de la Santé et de la Recherche Médicale (INSERM), National Research Institute for Agriculture, Food and Environment, Centre of Research in Epidemiology and Statistics, Paris, France; 10 Bradford Institute for Health Research, Bradford Teaching Hospitals NHS (National Health Service) Foundation Trust, Bradford, United Kingdom; 11 Department of Social Medicine, University of Crete, Heraklion, Greece; 12 Team of Environmental Epidemiology Applied to Reproduction and Respiratory Health, Institute for Advanced Biosciences, Grenoble Alpes University, INSERM, Centre National de la Recherche Scientiﬁque, La Tronche, France; 13 Clinical Biochemistry Department, Clínica Universidad de Navarra, Pamplona, Spain; 14 Department of Pediatrics, Emory University, Atlanta, Georgia

## Abstract

**Importance:**

Prenatal exposures to endocrine-disrupting chemicals (EDCs) may increase the risk for liver injury in children; however, human evidence is scarce, and previous studies have not considered potential EDC-mixture effects. Furthermore, the association between prenatal EDC exposure and hepatocellular apoptosis in children has not been studied previously.

**Objective:**

To investigate associations of prenatal exposure to EDC mixtures with liver injury risk and hepatocellular apoptosis in childhood.

**Design, Setting, and Participants:**

This prospective cohort study used data collected from April 1, 2003, to February 26, 2016, from mother-child pairs from the Human Early-Life Exposome project, a collaborative network of 6 ongoing, population-based prospective birth cohort studies from 6 European countries (France, Greece, Lithuania, Norway, Spain, and the UK). Data were analyzed from April 1, 2021, to January 31, 2022.

**Exposures:**

Three organochlorine pesticides, 5 polychlorinated biphenyls, 2 polybrominated diphenyl ethers (PBDEs), 3 phenols, 4 parabens, 10 phthalates, 4 organophosphate pesticides, 5 perfluoroalkyl substances, and 9 metals.

**Main Outcomes and Measures:**

Child serum levels of alanine aminotransferase (ALT), aspartate aminotransferase (AST), γ-glutamyltransferase (GGT), and CK-18 were measured at 6 to 11 years of age. Risk for liver injury was defined as having ALT, AST, and/or GGT levels above the 90th percentile. Associations of liver injury or cytokeratin 18 (CK-18) levels with each chemical group among the 45 EDCs measured in maternal blood or urine samples collected in pregnancy were estimated using 2 complimentary exposure-mixture methods: bayesian weighted quantile sum (BWQS) and bayesian kernel machine regression.

**Results:**

The study included 1108 mothers (mean [SD] age at birth, 31.0 [4.7] years) and their singleton children (mean [SD] age at liver assessment, 8.2 [1.6] years; 598 [54.0%] boys). Results of the BWQS method indicated increased odds of liver injury per exposure-mixture quartile increase for organochlorine pesticides (odds ratio [OR], 1.44 [95% credible interval (CrI), 1.21-1.71]), PBDEs (OR, 1.57 [95% CrI, 1.34-1.84]), perfluoroalkyl substances (OR, 1.73 [95% CrI, 1.45-2.09]), and metals (OR, 2.21 [95% CrI, 1.65-3.02]). Decreased odds of liver injury were associated with high-molecular-weight phthalates (OR, 0.74 [95% CrI, 0.60-0.91]) and phenols (OR, 0.66 [95% CrI, 0.54-0.78]). Higher CK-18 levels were associated with a 1-quartile increase in polychlorinated biphenyls (β, 5.84 [95% CrI, 1.69-10.08] IU/L) and PBDEs (β, 6.46 [95% CrI, 3.09-9.92] IU/L). Bayesian kernel machine regression showed associations in a similar direction as BWQS for all EDCs and a nonlinear association between phenols and CK-18 levels.

**Conclusions and Relevance:**

With a combination of 2 state-of-the-art exposure-mixture approaches, consistent evidence suggests that prenatal exposures to EDCs are associated with higher risk for liver injury and CK-18 levels and constitute a potential risk factor for pediatric nonalcoholic fatty liver disease.

## Key Points

**Question:**

Is prenatal exposure to endocrine-disrupting chemicals (EDCs) associated with liver injury and hepatocellular apoptosis in school-aged children?

**Findings:**

In the Human Early-Life Exposome population-based cohort study of 1108 mother-child pairs from 6 European countries, prenatal exposures to EDC mixtures, including organochlorine pesticides, polybrominated diphenyl ethers, perfluoroalkyl substances, and metals, significantly increased the risk for liver injury and/or hepatocellular apoptosis in school-aged children.

**Meaning:**

These findings suggest that exposure to mixtures of EDCs during the sensitive pregnancy period may increase the risk for liver injury and hepatocellular apoptosis in childhood, potentially contributing to the current epidemic of pediatric nonalcoholic fatty liver disease.

## Teaser

This population-based multinational cohort study investigates the association of the prenatal exposure to endocrine-disrupting chemicals with pediatric liver injury and hepatocellular apoptosis among mother-child pairs.

## Introduction

Nonalcoholic fatty liver disease (NAFLD) is one of the most common hepatic diseases worldwide and a major cause of extrahepatic comorbidities and hepatic transplantation.^1^ Nonalcoholic fatty liver disease is increasingly diagnosed in childhood,^2^ affecting 6% to 10% of the general pediatric population and approximately 34% of children with obesity.^3^ Growing evidence from animal and human studies shows that NAFLD programming may begin in utero.^4^ Early life exposures to endocrine-disrupting chemicals (EDCs) can affect liver development and metabolic programming in the fetus through hormone and epigenetic alterations, leading to long-term hepatotoxic effects,^5^ likely in combination with other established NAFLD risk factors such as genetic variations,^6^ diet,^7^ and obesity.^8^ Endocrine-disrupting chemicals are a wide class of environmental pollutants that interfere with hormone and metabolic systems in humans,^9^ including pesticides, plasticizers, toxic metals, and many other chemicals used in commercial and industrial applications.^10^ Several experimental studies have shown that exposures to EDCs can alter lipid influx-efflux balance in the liver and promote hormonal and mitochondrial dysfunction, hepatic inflammation and steatosis, liver injury, and NAFLD.^5,11^ However, the potential effects of EDC exposures in NAFLD are currently understudied in humans.^11,12^

Epidemiological evidence on the link between EDC exposure and NAFLD is scarce and mostly limited to cross-sectional studies in adults^11,13,14^ and a few studies in children.^15,16,17,18^ Previous studies of children^16,17,18,19^ relied on NAFLD assessment of serum levels of liver enzymes (eg, alanine aminotransferase [ALT]) that are established biomarkers for pediatric liver injury and NAFLD screening. Blood caspase–generated cytokeratin 18 (CK-18) fragment is a novel marker of hepatocyte apoptosis and NAFLD in children,^20^ but to our knowledge, no previous study has evaluated the association of prenatal EDC exposures with CK-18 levels. Furthermore, all previous studies have examined associations of individual chemicals or a particular group of EDCs with serum liver enzymes, but none has considered potential EDC mixture effects. Therefore, we investigated associations of prenatal exposure to 45 EDCs and EDC mixtures with risk for liver injury and serum CK-18 levels in children. We hypothesized that higher levels of prenatal exposure to EDC mixtures would be associated with increased odds of liver injury and hepatocellular apoptosis. To test our hypothesis, we used 2 complimentary state-of-the-art statistical approaches for exposure-mixture analysis, with each approach having its own specific strengths and limitations. To our knowledge, this is the most comprehensive investigation on the association of the prenatal exposome (as defined by environmental chemical biomarkers) with pediatric liver injury and hepatocellular apoptosis to date.

## Methods

### Study Population

The Human Early-Life Exposome (HELIX) project^21^ is a collaborative network of 6 established, ongoing, longitudinal population-based birth cohort studies in Europe: the Born in Bradford cohort in Bradford, UK^22^; the Étude des Déterminants Pré et Postnatals du Développement et de la Santé de l’Enfant cohort in France^23^; the Infancia y Medio Ambiente cohort in Spain^24^; the Kaunas cohort in Lithuania^25^; the Norwegian Mother, Father and Child Cohort Study^26^; and the RHEA Mother Child Cohort in Crete, Greece.^27^ A detailed description of the HELIX protocol is provided elsewhere.^28^ Data used in the present analyses were collected from April 1, 2003, to February 26, 2016. From 2013 to 2016, a subcohort of 1301 mothers and their singleton children in the HELIX project were followed up at children’s ages 6 to 11 years using the same research protocol, including clinical examination, interview with mothers, and biological samples collection^28^ (eligibility criteria for inclusion in the subcohort are provided in the eMethods in the Supplement). The present study included 1108 mother and children pairs (85.2%) from the HELIX subcohort who had measured EDC exposures during pregnancy and liver biomarkers in childhood. Self-reported data on race and ethnicity of the children were collected from the mothers in all HELIX subcohorts to facilitate comparisons with other study populations with respect to this European pediatric population. Written informed consent was provided by all participating families. Research protocol approval was obtained from local ethical committees at each site. In addition, this study was approved by the University of Southern California institutional review board. This study followed the Strengthening the Reporting of Observational Studies in Epidemiology (STROBE) reporting guideline.

### Exposure Assessment

Levels of 45 EDCs prioritized based on prior literature from animal and human studies supporting their role as metabolic disruptors^11,28,29^ were measured in maternal blood or urine samples collected in pregnancy or cord blood collected at birth by individual HELIX subcohorts as detailed elsewhere^28,29,30^ (eTables 1 and 2 in the Supplement). The EDCs included 3 organochlorine pesticides, 5 polychlorinated biphenyls (PCBs), 2 polybrominated diphenyl ethers (PBDEs), 3 phenols, 4 parabens, 10 phthalates, 4 organophosphate pesticides, 5 perfluoroalkyl substances (PFASs), and 9 metals. Levels of all persistent organic pollutants (ie, organochlorine pesticides, PCBs, PBDEs, and PFASs) and metals were measured in maternal blood in all cohorts, except for mercury, which was measured in cord blood for the Infancia y Medio Ambiente cohort.^30^ Persistent organic pollutant levels with known lipophilic properties (organochlorine pesticides, PCBs, and PBDEs) were corrected for blood lipid content and are expressed in nanograms per gram of lipid. Nonpersistent EDC levels (phthalates, phenols, and organophosphate pesticides) were measured in a 1-spot maternal urine sample,^30^ and concentrations were corrected for urine creatinine levels to adjust for urine dilution. Measured phthalate urine metabolites included high-molecular-weight phthalates (molecular mass ≥250 kDa), mono-benzyl phthalate, 4 metabolites of parent compound diethylhexyl phthalate (mono-2-ethylhexyl phthalate, mono[2-ethyl-5-oxohexyl] phthalate, mono[2-ethyl-5-hydroxyhexyl] phthalate, and methylerythritol cyclodiphosphate), 2 metabolites of parent compound Di-iso-nonylphthalate (mono-oxo-isononyl phthalate and mono-hydroxy-isononyl phthalate), and 3 low-molecular-weight phthalates (molecular mass <250 kDa).^31^

### Outcome Assessment

As primary outcomes, we analyzed risk for liver injury (based on ALT, aspartate aminotransferase [AST], and γ-glutamyltransferase [GGT] serum levels) and CK-18 serum levels as a marker of hepatocellular apoptosis. All biomarkers were measured using standard assays in child serum samples at ages 6 to 11 years, as detailed elsewhere^17^ (eMethods in the Supplement). In the absence of universally accepted liver enzyme cutoff levels to define liver injury and NAFLD in children,^32,33^ and because the HELIX study includes a population-based cohort of apparently healthy children, we defined risk of liver injury as having any of the liver enzyme concentrations above the 90th percentile of the study population (ie, ALT ≥22.7 IU/L; AST ≥41.4 IU/L; and GGT ≥17.1 IU/L [to convert liver enzyme levels to microkatals per liter, multiply by 0.0167]).^17^ Additional sensitivity analyses were performed using liver enzyme outcome variables continuously.

### Statistical Analysis

#### Statistical Models

Data were analyzed from April 1, 2021, to January 31, 2022. We used triangulation of state-of-the-art statistical methods for exposure-mixture analysis to confirm the robustness of associations independently of the statistical approach and identify consistent findings across methods because each method presents unique strengths and limitations (eTable 3 in the Supplement). To facilitate comparison of estimates across statistical methods, all chemical exposures were converted to quartiles. Primary statistical analyses included 2 approaches for the evaluation of the associations of EDCs as mixtures. First, we used bayesian weighted quantile sum (BWQS) regressions that assume linear and additive effects^34^ to estimate associations of all EDCs belonging to a particular group with the study outcomes. Second, we used bayesian kernel machine regression (BKMR) that relaxes the assumptions of linearity and additivity^35^ to identify nonlinear EDC group associations. Furthermore, we conducted a secondary sensitivity analysis analyzing 1 chemical at a time, an approach that has been almost exclusively applied in this field previously, and compared the results with those obtained from the 2 exposure-mixture approaches. For individual-chemical analyses, we used generalized linear mixed-effect regression models with a 2-tailed *P* < .05 as the level of significance and controlled for multiple comparison error by calculating *q* values. Analyses were conducted using R, version 4.0.3 (R Project for Statistical Computing). A schematic diagram of the statistical analysis plan and more details are provided in eFigure 5 and the eMethods in the Supplement.

#### Covariates

All statistical models were adjusted for the following confounders and variables associated with outcomes, selected based on a priori knowledge and directed acyclic graphs^36^ (eFigure 1 in the Supplement): subcohort, maternal age, maternal prepregnancy body mass index (BMI [calculated as weight in kilograms divided by height in meters squared]), maternal educational level,^37^ parity, and child age and sex. We adjusted for child BMI only in sensitivity analyses but not in main analyses, because prenatal EDC exposures have been associated with child BMI,^31,38^ and therefore BMI may be a mediating factor in the association between prenatal EDC exposures and liver outcomes.^39^

#### Sensitivity Analysis

We conducted the following sensitivity analyses to evaluate the robustness of results. First, we performed individual-chemical analyses using generalized linear mixed-effect regression (as described above). Second, we evaluated EDC associations with each liver enzyme level (ALT, AST, or GGT) separately, controlling for confounders. Third, we tested for effect modification by sex and stratified analysis of liver injury and CK-18 levels by sex because previous studies have shown sexually dimorphic metabolic effects of prenatal EDC exposures.^31,40^ Fourth, we examined whether the timing of maternal spot urine collection (second or third pregnancy trimester) modifies the associations with liver outcomes observed for short half-life EDCs measured in urine. Fifth, we repeated analyses by adjusting the statistical models further for postnatal EDC exposures or maternal diet (consumption of fish, fruits, and vegetables in times per week) to evaluate confounding by these factors in the EDC associations with liver outcomes. Fish, fruits, and vegetables are sources of EDC exposure in pregnant women.^41,42^ We also adjusted the statistical models for child BMI *z* scores.

## Results

**Figure 1.**
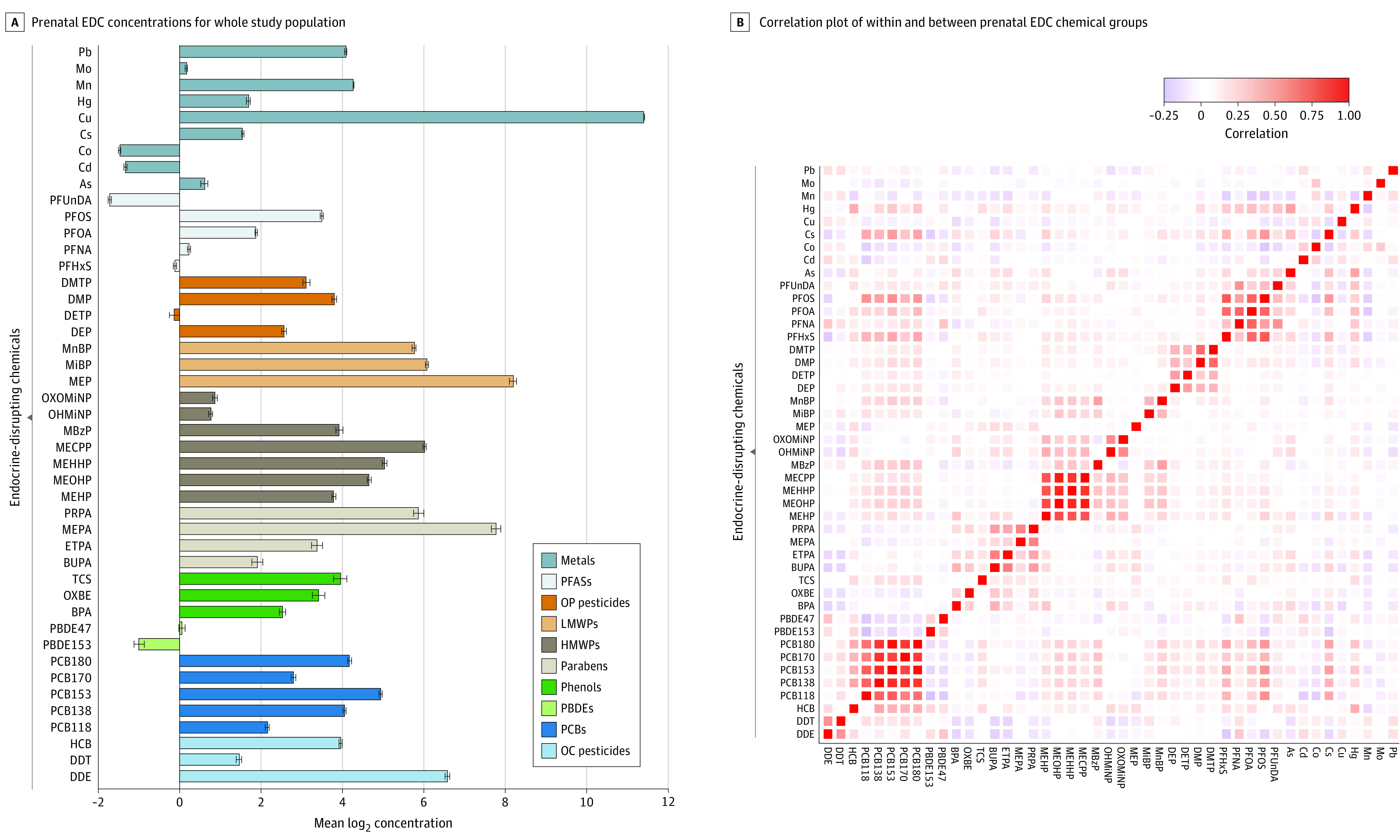
Prenatal Endocrine-Disrupting Chemical (EDC) Concentrations and Correlation Plot Error bars indicate 2*SE log (base = 2) concentration. As indicates inorganic arsenic; BPA, bisphenol A; BUPA, N-butyl paraben; Cd, cadmium; Co, cobalt; Cs, caesium; Cu, copper; DDE, dichlorodiphenyldichloroethylene; DDT, dichlorodiphenyltrichloroethane; DEP, diethyl phthalate; DETP, diethyl thiophosphate; DMP, dimethyl phthalate; DMTP, dimethylthiophosphate; ETPA, ethyl paraben; HCB, hexachlorobenzene; Hg, mercury; HMWPs, high-molecular-weight phthalates; LMWPs, low-molecular-weight phthalates; MBzP, monobenzylphthalate; MECPP, methylerythritol cyclodiphosphate; MEHHP, mono(2-ethyl-5-hydroxyhexyl) phthalate; MEHP, mono-2-ethylhexyl phthalate; MEOHP, mono(2-ethyl-5-oxohexyl) phthalate; MEP, monoethyl phthalate; MEPA, methyl paraben; MiBP, mono-iso-butyl phthalate; Mn, manganese; MnBP, mono-n-butyl phthalate; Mo, molybdenum; OC, organochlorine; OHMiNP, mono-hydroxy-isononyl phthalate; OP, organophosphate; OXBE, oxybenzone; OXOMiNP, mono-oxo-isononyl phthalate; Pb, lead; PBDEs, polybrominated diphenyl ethers; PCBs, polychlorinated biphenyls; PFASs, perfluoroalkyl substances; PFHxS, perfluorohexane sulfonate; PFNA, perfluorononanoic acid; PFOA, perfluoro-octanoic acid; PFOS, perfluoro-octane sulfonate; PFUnDA, perfluoroundecanoic acid; PRPA, propyl paraben; and TCS, triclosan.

We studied a total of 1108 children, of whom 253 (22.8%) were classified as being at high risk for liver injury; the highest prevalence was in children from Greece (80 of 253 [31.6%]) and the lowest prevalence in children from Lithuania (11 of 253 [4.3%]). Mean (SD) maternal age at birth was 31.0 (4.7) years. Among the children, mean (SD) age at liver assessment was 8.2 (1.6) years; 510 (46.0%) were girls and 598 (54.0%) were boys. The Table presents the characteristics of mothers and children in the HELIX project to facilitate comparisons with other study populations and includes age- and sex-specific BMI *z* scores from the World Health Organization growth reference.^43^ Children at risk for liver injury were more likely to be overweight or obese (87 of 253 [34.4%] vs 144 of 855 [16.8%]) and members of racial and ethnic minority groups (40 of 253 [15.8%] vs 37 of 855 [4.3%]), and their mothers had lower educational status (40 of 253 [15.8%] vs 84 of 855 [9.8%]) and higher mean (SD) BMI (25.8 [5.7] vs 24.2 [4.5]). Prenatal EDC exposure concentrations are presented in Figure 1A and eTable 4 in the Supplement. Significant Pearson correlations between EDC pairs were mostly positive and ranged from low (0.10) to high (0.80). A few low to moderate negative correlations (from −0.10 to −0.25) were also observed between EDCs (Figure 1B).

**Table.** Characteristics of Mothers and Children in the HELIX Project, Overall and by Liver Injury Risk

Characteristic	Liver injury risk group^a^			*P* value for difference^b^
	All (N = 1108)	Low (n = 855)	High (n = 253)	
**Subcohort**				
BiB (UK)	101 (9.1)	45 (5.3)	56 (22.1)	<.001
EDEN (France)	190 (17.1)	150 (17.5)	40 (15.8)	
INMA (Spain)	215 (19.4)	166 (19.4)	49 (19.4)	
KANC (Lithuania)	170 (15.3)	159 (18.6)	11 (4.3)	
MoBa (Norway)	268 (24.2)	251 (29.3)	17 (6.7)	
RHEA (Greece)	164 (14.8)	84 (9.8)	80 (31.6)	
**Maternal**				
Age at birth, mean (SD), y	31.0 (4.7)	31.1 (4.7)	31.0 (4.8)	.97
Prepregnancy BMI, mean (SD)	24.5 (4.8)	24.2 (4.5)	25.8 (5.7)	<.001
Educational level^c^				
Low	124 (11.2)	84 (9.8)	40 (15.8)	<.001
Medium	383 (34.6)	278 (32.5)	105 (41.5)	
High	601 (54.2)	493 (57.7)	108 (42.7)	
Parity				
Nulliparous	507 (45.7)	397 (46.4)	110 (43.5)	.09
Primiparous	407 (36.7)	320 (37.4)	87 (34.4)	
Multiparous	194 (17.5)	138 (16.1)	56 (22.1)	
**Child**				
Age at liver assessment, mean (SD), y	8.2 (1.6)	8.3 (1.6)	7.8 (1.7)	<.001
Sex				
Female	510 (46.0)	398 (46.5)	112 (44.3)	.57
Male	598 (54.0)	457 (53.5)	141 (55.7)	
Race and ethnicity				
White	1031 (93.1)	818 (95.7)	213 (84.2)	<.001
Other^d^	77 (6.9)	37 (4.3)	40 (15.8)	
BMI, mean (SD)	17.0 (2.6)	16.7 (2.2)	17.9 (3.5)	<.001
BMI *z* scores, mean (SD)^e^	0.4 (1.2)	0.3 (1.1)	0.8 (1.5)	<.001
Overweight or obesity^e^	231 (20.8)	144 (16.8)	87 (34.4)	<.001
Liver outcome concentrations, mean (SD), IU/L				
ALT	15.7 (6.3)	13.7 (3.7)	22.3 (8.6)	<.001
AST	30.9 (9.3)	28.2 (5.1)	39.7 (13.6)	<.001
GGT	12.8 (4.9)	11.7 (2.2)	16.6 (8.4)	<.001
CK-18	82.0 (48.6)	82.2 (50.1)	81.3 (42.9)	.79

Abbreviations: ALT, alanine aminotransferase; AST, aspartate aminotransferase; BiB, Born in Bradford; BMI, body mass index (calculated as weight in kilograms divided by height in meters squared); CK-18, cytokeratin 18; EDEN, Étude des Déterminants Pré et Postnatals du Développement et de la Santé de l’Enfant; GGT, γ-glutamyltransferase; HELIX, Human Early-Life Exposome; INMA, Infancia y Medio Ambiente; KANC, Kaunas cohort; MoBa, Norwegian Mother, Father and Child; RHEA, RHEA Mother Child Cohort.

SI conversion factors: To convert ALT, AST, CK-18, and GGT to microkatals per liter, multiply by 0.0167.

^a^ Liver injury risk was defined as having any liver enzyme level (ALT, AST, or GGT) above the 90th percentile. Unless otherwise indicated, data are expressed as No. (%) of participants. Percentages have been rounded and may not total 100.

^b^ *P* values for difference between the low- and high-risk groups for liver injury were calculated using the Fisher exact test for categorical variables and Wilcoxon rank-sum test for continuous variables.

^c^ Defined using the International Standard Classification of Education.

^d^ Includes Asian, Black, Native American or Alaska Native, and multiple races and/or ethnicities.

^e^ Age- and sex-specific BMI *z* scores and overweight or obese were calculated using the World Health Organization growth reference.^43^

### BWQS Results

**Figure 2.**
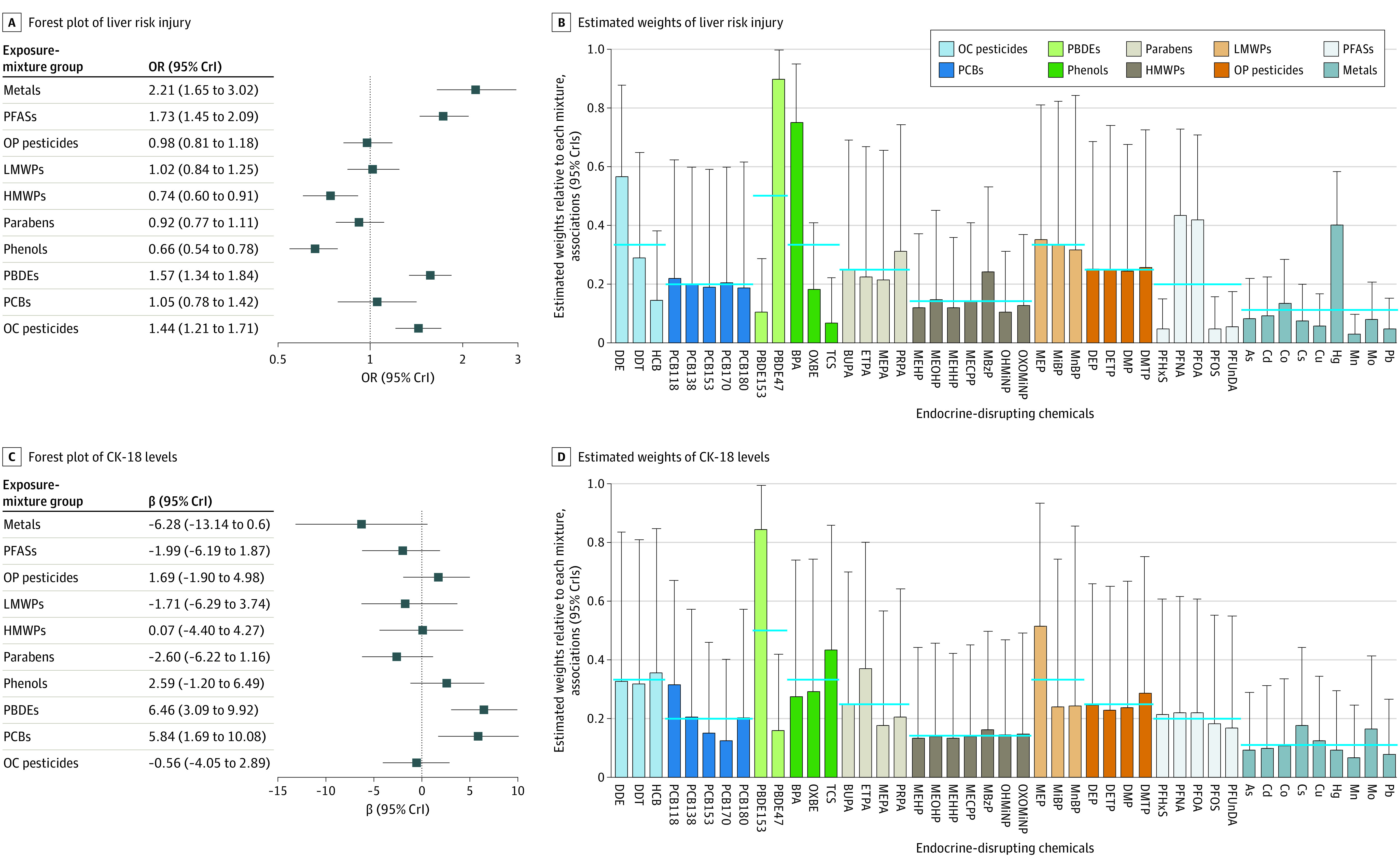
Forest Plots and Estimated Posterior Weights of Exposure-Mixture Groups on Liver Injury and Cytokeratin 18 (CK-18) Levels Using the Bayesian Weighted Quantile Sum Method Within an exposure-mixture group, the estimated weights total 1, implying relative contribution of each exposure to the overall group association. Blue horizontal lines for the estimated weights denote expected weights if all chemicals within a group contributed equally to the exposure mixture. All models were adjusted for subcohort, maternal age, maternal prepregnancy body mass index, maternal educational level, parity, child sex, and child age. As indicates inorganic arsenic; BPA, bisphenol A; BUPA, N-butyl paraben; Cd, cadmium; Co, cobalt; Cs, caesium; Cu, copper; DDE, dichlorodiphenyldichloroethylene; DDT, dichlorodiphenyltrichloroethane; DEP, diethyl phthalate; DETP, diethyl thiophosphate; DMP, dimethyl phthalate; DMTP, dimethylthiophosphate; ETPA, ethyl paraben; HCB, hexachlorobenzene; Hg, mercury; HMWPs, high-molecular-weight phthalates; LMWPs, low-molecular-weight phthalates; MBzP, monobenzylphthalate; MECPP, methylerythritol cyclodiphosphate; MEHHP, mono(2-ethyl-5-hydroxyhexyl) phthalate; MEHP, mono-2-ethylhexyl phthalate; MEOHP, mono(2-ethyl-5-oxohexyl) phthalate; MEP, monoethyl phthalate; MEPA, methyl paraben; MiBP, mono-iso-butyl phthalate; Mn, manganese; MnBP, mono-n-butyl phthalate; Mo, molybdenum; OC, organochlorine; OHMiNP, mono-hydroxy-isononyl phthalate; OP, organophosphate; OXBE, oxybenzone; OXOMiNP, mono-oxo-isononyl phthalate; Pb, lead; PBDEs, polybrominated diphenyl ethers; PCBs, polychlorinated biphenyls; PFASs, perfluoroalkyl substances; PFHxS, perfluorohexane sulfonate; PFNA, perfluorononanoic acid; PFOA, perfluoro-octanoic acid; PFOS, perfluoro-octane sulfonate; PFUnDA, perfluoroundecanoic acid; PRPA, propyl paraben; and TCS, triclosan.

An association with increased odds of liver injury per 1-quartile increase in EDC group mixture was found for organochlorine pesticides (odds ratio [OR], 1.44 [95% credible interval (CrI), 1.21-1.71]), PBDEs (OR, 1.57 [95% CrI, 1.34-1.84]), PFASs (OR, 1.73 [95% CrI, 1.45-2.09]), and metals (OR, 2.21 [95% CrI, 1.65-3.02]). An association with decreased odds of liver injury was found for phenols (OR, 0.66 [95% CrI, 0.54-0.78]) and high-molecular-weight phthalates (OR, 0.74 [95% CrI, 0.60-0.91]) (Figure 2A and eTables 5 and 6 in the Supplement). The highest contributor in the PBDE mixture association with liver injury was PBDE47, whereas perfluorononanoic acid and perfluoro-octanoic acid were the highest contributors in the PFAS mixture association (Figure 2B). Among metals and phenols, mercury and bisphenol A, respectively, were the highest contributors, but the weights were not statistically significantly higher compared with other chemicals within their group.

A 1-quartile increase in PBDE and PCB exposure was associated with increased CK-18 levels (β = 6.46 [95% CrI, 3.09-9.92] IU/L and β = 5.84 [95% CrI, 1.69-10.08] IU/L, respectively). The major contributor to the PBDE mixture association was PBDE153, whereas PCB118 had nonsignificantly higher weight (β = 0.32 [95% CrI, 0.03-0.67]) among PCBs (PCB138, β = 0.21 [95% CrI, 0.01-0.57]; PCB180, β = 0.20 [95% CrI, 0.01-0.57]) (Figure 2C and D). We observed no association with liver injury or CK-18 levels for prenatal exposures to organophosphate pesticides, parabens, and low-molecular-weight phthalates.

### BKMR Results

**Figure 3.**
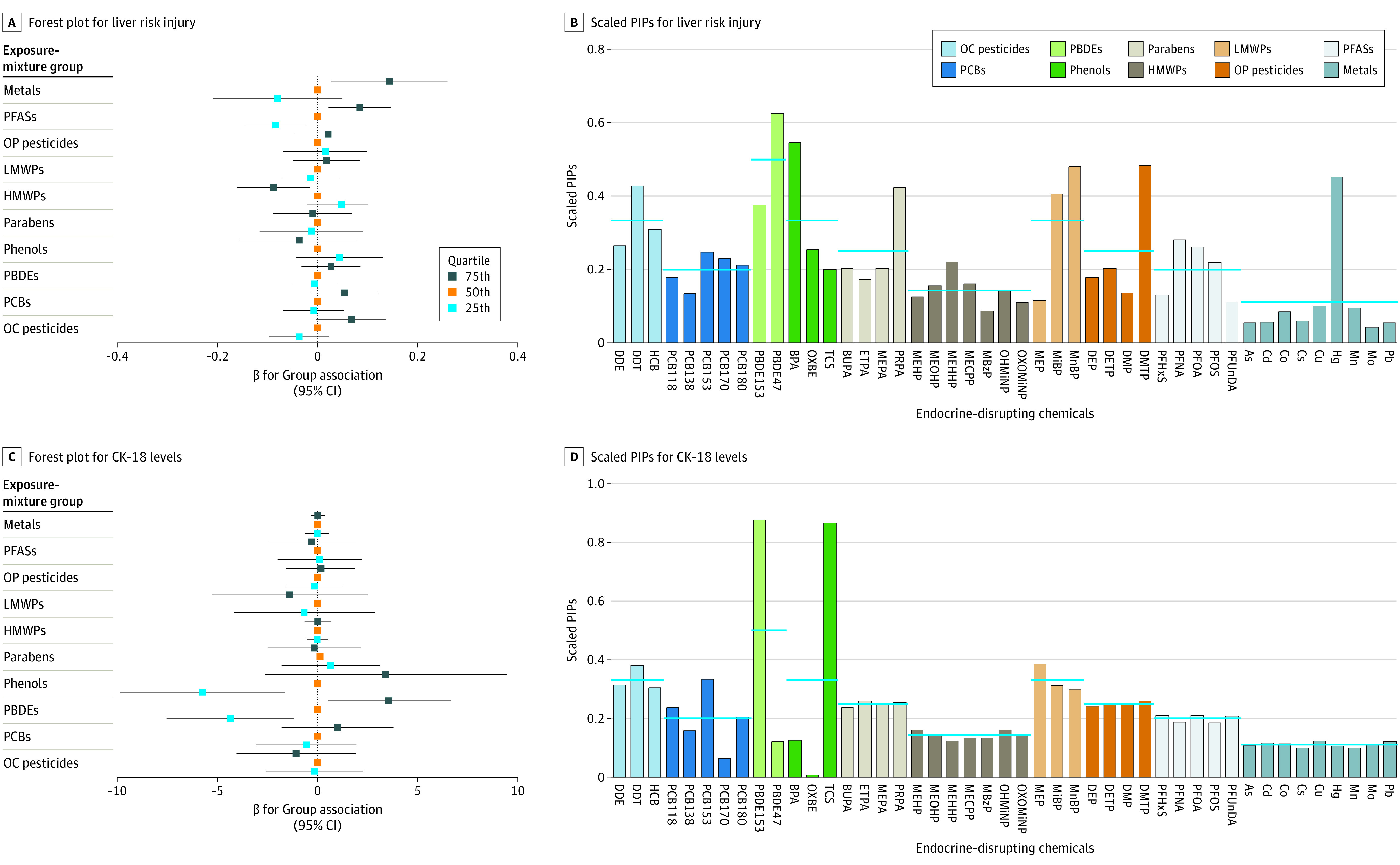
Forest Plots and Scaled Posterior Inclusion Probabilities (PIPs) of Exposure-Mixture Groups for Liver Injury and Cytokeratin 18 (CK-18) Levels Using the Bayesian Kernel Machine Regression (BKMR) Method Blue horizontal lines in the BKMR models denote expected scaled PIPs if all chemicals within a group were included equally while estimating mixture associations. As indicates inorganic arsenic; BPA, bisphenol A; BUPA, N-butyl paraben; Cd, cadmium; Co, cobalt; Cs, caesium; Cu, copper; DDE, dichlorodiphenyldichloroethylene; DDT, dichlorodiphenyltrichloroethane; DEP, diethyl phthalate; DETP, diethyl thiophosphate; DMP, dimethyl phthalate; DMTP, dimethylthiophosphate; ETPA, ethyl paraben; HCB, hexachlorobenzene; Hg, mercury; HMWPs, high-molecular-weight phthalates; LMWPs, low-molecular-weight phthalates; MBzP, monobenzylphthalate; MECPP, methylerythritol cyclodiphosphate; MEHHP, mono(2-ethyl-5-hydroxyhexyl) phthalate; MEHP, mono-2-ethylhexyl phthalate; MEOHP, mono(2-ethyl-5-oxohexyl) phthalate; MEP, monoethyl phthalate; MEPA, methyl paraben; MiBP, mono-iso-butyl phthalate; Mn, manganese; MnBP, mono-n-butyl phthalate; Mo, molybdenum; OC, organochlorine; OHMiNP, mono-hydroxy-isononyl phthalate; OP, organophosphate; OXBE, oxybenzone; OXOMiNP, mono-oxo-isononyl phthalate; Pb, lead; PBDEs, polybrominated diphenyl ethers; PCBs, polychlorinated biphenyls; PFASs, perfluoroalkyl substances; PFHxS, perfluorohexane sulfonate; PFNA, perfluorononanoic acid; PFOA, perfluoro-octanoic acid; PFOS, perfluoro-octane sulfonate; PFUnDA, perfluoroundecanoic acid; PRPA, propyl paraben; and TCS, triclosan.

Differences in effect sizes for liver injury risks when all the exposures in an EDC group were at a particular percentile (25th or 75th) with respect to when all were fixed at the 50th percentile are shown in Figure 3 and in eTables 7 and 8 in the Supplement. A 1-quartile increase (from the 50th to 75th percentile) in prenatal exposure was associated with increased liver injury for organochlorine pesticides (β = 0.07 [95% CrI, −0.00 to 0.14]), PBDEs (β = 0.03 [95% CrI, −0.03 to 0.09]), PFASs (β = 0.08 [95% CrI, 0.02-0.15]), and metals (β = 0.14 [95% CrI, 0.03-0.26]). We found an association with decreased liver injury for prenatal exposures to phenols (β = −0.04 [95% CrI, −0.15 to 0.08]) and high-molecular-weight phthalates (β = −0.09 [95% CrI, −0.16 to −0.02]) (Figure 3A). In agreement with BWQS results, PBDE47, bisphenol A, perfluorononanoic acid, perfluoro-octanoic acid, and mercury had the highest scaled posterior inclusion probabilities within their EDC group mixture (Figure 3B).

A 1-quartile increase in prenatal exposure was associated with increased CK-18 levels for PCBs (β = 0.98 [95% CrI, −1.82 to 3.79] IU/L and PBDEs (β = 3.58 [95% CrI, 0.51-6.64] IU/L) (from the 50th to 75th percentile) (Figure 3C). Within PBDEs, PBDE153 had the highest scaled posterior inclusion probabilities, whereas PCB118, PCB153, and PCB180 were the highest contributors among PCBs. In addition to BWQS results, BKMR analysis suggested a nonlinear association between phenols and increased CK-18 levels (from 50th to 25th percentile, β = −5.74 IU/L [95% CrI, −9.85 to −1.63] IU/L; from 50th to 75th percentile, β = 3.40 [95% CrI, −2.62 to 9.42] IU/L) (Figure 3C), with triclosan having the highest scaled posterior inclusion probabilities (Figure 3D).

### Sensitivity Analysis

We observed individual-chemical associations in the same direction as the associations of EDC group mixtures, but only a few associations were statistically significant (eFigure 2, eResults, and eTable 9 in the Supplement). The sign of the associations of EDCs with each individual liver enzyme (ALT, AST, and GGT) remained similar to the associations observed for combined liver injury outcome (eFigure 3 and eTable 10 in the Supplement). Organochlorine pesticides and metals were more strongly associated with odds of liver injury in male participants compared with female participants, but no significant sex interactions were observed between EDCs and CK-18 levels (eFigure 3, eResults, and eTable 11 in the Supplement). The different timing of maternal spot urine collection across cohorts did not change the associations between nonpersistent EDCs and liver outcomes (eFigure 4 in the Supplement). Effect estimates did not meaningfully change after controlling for postnatal EDC exposures, maternal diet, or child BMI *z* scores (eResults and eTables 12-14 in the Supplement).

## Discussion

In this multicenter cohort study of mothers and their children from Europe, we found evidence that prenatal exposures to organochlorine pesticides, PBDEs, PFASs, and metals were associated with increased liver injury risk in children, and exposure to PBDEs and PCBs were further associated with increased CK-18 levels. We corroborated these associations using 2 state-of-the-art statistical methods available for exposure-mixture analysis, thus providing more robust evidence for an association between prenatal exposure to EDCs and liver injury and hepatocellular apoptosis in childhood. Bayesian kernel machine regression analysis further indicated a nonlinear association between phenols and CK-18 levels that was mainly driven by exposure to triclosan. We observed null associations independently of the statistical approach used for exposure to parabens, low-molecular-weight phthalates, and organophosphate pesticides.

Organochlorine pesticides and PBDEs are persistent, lipophilic chemicals that cross the placental barrier and may disrupt fetal metabolic programing.^44^ Maternal serum concentrations of dichlorodiphenyldichloroethylene and PBDEs in our study were lower compared with US National Health and Nutrition Examination Survey (NHANES) concentrations among women from 1999 to 2010,^45^ when mothers from the HELIX project were enrolled. Previous studies have reported associations of higher prenatal exposure to organochlorine pesticides and PBDEs with higher BMI, overweight risk, and/or serum lipid levels in school-aged children.^46^ Findings from the present analysis advance the state of evidence by showing that exposure to mixtures of organochlorine pesticides and PBDEs may increase the risk for pediatric liver injury. In agreement with our findings, a recent study using data from US 2003-2004 NHANES reported associations between organochlorine pesticides and elevated ALT levels in adults.^47^ Similarly, another US NHANES study^48^ reported significant positive associations between serum concentrations of PBDE153 and alkaline phosphatase levels but no association with other liver enzymes. We observed a 2- to 10-IU/L mean increase in CK-18 levels per quartile increases in PBDEs and PCBs. Although we cannot confirm the clinical relevance of these findings in the HELIX study, a previous study of 201 children aged approximately 10 years with biopsy-proven NAFLD^49^ reported a 70% increase in odds of having nonalcoholic steatohepatitis for every 10-U/L increase in plasma CK-18. Thus, our findings may be clinically important and indicate a shift of the general pediatric population toward higher risks for nonalcoholic steatohepatitis.

Prenatal exposures to PFASs and mercury were previously associated with elevated ALT levels and liver injury in children in the HELIX project.^17,18^ Previous cross-sectional studies^16,50^ reported positive associations between the plasma or serum concentration of PFASs, including perfluoro-octanoic acid, perfluoro-octane sulfonate, perfluorononanoic acid, and perfluorohexane sulfonate, and liver enzyme levels and/or nonalcoholic steatohepatitis in US children. Similarly, among metals, mercury exposure had been linked to elevated ALT levels in adolescents in the US NHANES^51^ and increased ALT and GGT levels in adults from South Korea.^52^ A strength of our analysis is that we considered a mixture of metals and identified mercury as the potential main driver of the metal mixture association with liver injury.

Phenols, parabens, phthalates, and organophosphate pesticides are nonpersistent chemicals metabolized and excreted rapidly (within hours or days) from the body.^53^ Previous evidence in rodents supports a link between perinatal bisphenol A exposure and steatohepatitis,^54^ as well as prenatal diethylhexyl phthalate exposure and liver damage.^55^ However, we observed mostly null associations with both liver injury and CK-18 levels for all nonpersistent chemicals examined, with the exception of negative associations observed for phenols and high-molecular-weight phthalates with liver injury and a nonlinear positive association between phenols and CK-18 levels.

### Strengths and Limitations

Our study has several strengths. First, we applied a novel analytical framework using 2 state-of-the-art statistical methods for exposure-mixture analysis with varying underlying assumptions that permitted us to assess the robustness of results, independent of the statistical approach. We further showed that exposure-mixture approaches may offer enhanced precision in identifying EDC associations with health outcomes compared with 1-chemical-at-a-time approaches that have been almost exclusively applied in this research field to date. Individuals are simultaneously exposed to complex EDC mixtures, and therefore exposure-mixture approaches are essential to characterize effects of EDCs on health. Second, this is a multicenter prospective study from 6 European cohorts with detailed assessment of multiple environmental chemical exposures in pregnancy that provide the most comprehensive evaluation of the association between the prenatal chemical exposome and pediatric liver injury and hepatocellular apoptosis to date, to our knowledge. Third, associations remained robust after adjusting for postnatal EDC exposures, further supporting pregnancy as a sensitive period for EDC effects on metabolic programming and NAFLD risk in children.

The limitations of this study include potential measurement error in the assessment of nonpersistent chemicals using spot urine samples, which may explain in part some of the null or negative associations observed for nonpersistent chemicals. Previous studies have also reported mixed results (positive, negative, and null associations) between prenatal phthalate exposures and childhood BMI, blood pressure, or serum lipid levels.^31,56^ Another explanation for discrepancies in phthalate findings across studies might be potential interactions by other prenatal exposures (eg, maternal diet) beyond the focus of the present study. Further studies with repeated over-time measurements of nonpersistent EDCs are needed to corroborate any association with NAFLD during the life course. We focused on a large list of measured EDCs with well-documented metabolic disrupting actions.^11,28,29^ However, other EDCs not measured in our study, such as emerging PFAS exposures, could also be hepatotoxic^57^ and warrant attention in future studies. One more limitation is the biomarker-based assessment for NAFLD instead of the criterion standard liver biopsy, which is unfeasible in large-scale, population-based studies of healthy children because of its risk and ethical limitations.^17^ Findings from this study show higher risk for pediatric liver injury associated with in utero EDC exposures, in line with previous experimental evidence,^11^ but cannot establish a link with NAFLD diagnosis per se, because this is a population-based cohort of relatively healthy and young children.

## Conclusions

In this population-based cohort study, we used state-of-the-art exposure-mixture approaches and found that prenatal exposures to ubiquitous EDCs, and especially to organochlorine pesticides, PBDEs, PFASs, and metals, were associated with increased liver injury and/or hepatocellular apoptosis in children. These results advance the current limited understanding of pediatric NAFLD etiology and support the need for more investigation in this area. Our findings can inform more efficient early-life prevention and intervention strategies to address the current NAFLD epidemic.

## Notes

Supplement. **eFigure 1.** Directed Acyclic Graphs (DAGs) Elucidating Exposure-Outcome Association **eFigure 2.** Forest Plots for Individual Chemical Exposures for Liver Injury and CK-18 Outcomes From Linear Mixed Models **eFigure 3.** Sensitivity Analyses of Individual Liver Enzymes (ALT, GGT, and AST) and Stratified Analyses by Child Sex Using BWQS **eFigure 4.** Sensitivity Analyses for Shorter Half-life Chemicals (Phenols, Parabens, Phthalates and OP Pesticides) Stratifying Between Those Cohorts Who Measured Exposures in the Second Pregnancy Trimester vs Third Trimester **eFigure 5.** Schematic Diagram of the Statistical Analysis Plan, Detailing the Sequences of the Analyses **eMethods.** Description of HELIX Subcohort and Inclusion Criteria, Liver Outcome Assessment, Details on Statistical Methods, and Postnatal EDC Exposure Assessment **eResults.** GLMER Results for Individual Chemicals and Results of Sensitivity Analyses **eTable 1.** Prenatal EDC Exposure Assessment **eTable 2.** Biological Matrices, Timing of Sample Collection, Laboratories, Analytical Methods, and Quality Controls for Prenatal EDC Exposures Assessment **eTable 3.** Statistical Approaches for Analysis of Multiple Correlated Exposures **eTable 4.** Log (Base = 2) Transformed Prenatal Blood and Serum Mean (SE) Concentrations of the Chemical Exposures **eTable 5.** BWQS Models for Liver Injury and CK-18 **eTable 6.** Estimated Posterior Weights of Exposure Mixture Groups on Liver Injury and CK-18 Outcomes Using BWQS Models **eTable 7.** BKMR Models for Liver Injury and CK-18 **eTable 8.** Estimated Posterior Inclusion Probabilities (PIPs) of Exposures in the Mixture Groups for Liver Injury and CK-18 Outcomes Using BKMR Models **eTable 9.** Linear Mixed Effects Regression (LMER) Models for Individual Chemicals on Binary Liver Injury and Continuous CK-18 Outcomes **eTable 10.** Analyses of Individual Liver Enzymes (ALT, AST, and GGT) Using BWQS Models **eTable 11.** Stratified BWQS Analyses of Liver Injury and CK-18 by Sex of the Children **eTable 12.** BWQS Analyses of Liver Injury and CK-18 After Adjusting for Postnatal Chemical Exposures **eTable 13.** BWQS Analyses of Liver Injury and CK-18 After Adjusting for Maternal Diet Quality (Consumption of Fish, Fruits, and Vegetables in Times per Week) **eTable 14.** BWQS Analyses of Liver Injury and CK-18 After Adjusting for Child BMI *z* Score
eReferences
