# Supplementary material for: Association of Prenatal Exposure to Endocrine-Disrupting Chemicals With Liver Injury in Children
Source: JAMA Netw Open. 2022 Jul 6;5(7):e2220176. doi: 10.1001/jamanetworkopen.2022.20176 (PMC9260485; doi:10.1001/jamanetworkopen.2022.20176)
Supplement: Supplement. — eFigure 1. Directed Acyclic Graphs (DAGs) Elucidating Exposure-Outcome Association eFigure 2. Forest Plots for Individual Chemical Exposures for Liver Injury and CK-18 Outcomes From Linear Mixed Models eFigure 3. Sensitivity Analyses of Individual Liver Enzymes (ALT, GGT, and AST) and Stratified Analyses by Child Sex Using BWQS eFigure 4. Sensitivity Analyses for Shorter Half-life Chemicals (Phenols, Parabens, Phthalates and OP Pesticides) Stratifying Between Those Cohorts Who Measured Exposures in the Second Pregnancy Trimester vs Third Trimester eFigure 5. Schematic Diagram of the Statistical Analysis Plan, Detailing the Sequences of the Analyses eMethods. Description of HELIX Subcohort and Inclusion Criteria, Liver Outcome Assessment, Details on Statistical Methods, and Postnatal EDC Exposure Assessment eResults. GLMER Results for Individual Chemicals and Results of Sensitivity Analyses eTable 1. Prenatal EDC Exposure Assessment eTable 2. Biological Matrices, Timing of Sample Collection, Laboratories, Analytical Methods, and Quality Controls for Prenatal EDC Exposures Assessment eTable 3. Statistical Approaches for Analysis of Multiple Correlated Exposures eTable 4. Log (Base = 2) Transformed Prenatal Blood and Serum Mean (SE) Concentrations of the Chemical Exposures eTable 5. BWQS Models for Liver Injury and CK-18 eTable 6. Estimated Posterior Weights of Exposure Mixture Groups on Liver Injury and CK-18 Outcomes Using BWQS Models eTable 7. BKMR Models for Liver Injury and CK-18 eTable 8. Estimated Posterior Inclusion Probabilities (PIPs) of Exposures in the Mixture Groups for Liver Injury and CK-18 Outcomes Using BKMR Models eTable 9. Linear Mixed Effects Regression (LMER) Models for Individual Chemicals on Binary Liver Injury and Continuous CK-18 Outcomes eTable 10. Analyses of Individual Liver Enzymes (ALT, AST, and GGT) Using BWQS Models eTable 11. Stratified BWQS Analyses of Liver Injury and CK-18 by Sex of the Children eTable 12. BWQS Analyses of Liver Inju [file jamanetwopen-e2220176-s001.pdf]

Supplementary Online Content

Midya V, Colicino E, Conti DV, et al. Association of prenatal exposure to endocrine-disrupting chemicals with liver injury in children. *JAMA Netw Open*. 2022;5(7):e2220176. doi:10.1001/jamanetworkopen.2022.20176

eFigure 1. Directed Acyclic Graphs (DAGs) Elucidating Exposure-Outcome Association
eFigure 2. Forest Plots for Individual Chemical Exposures for Liver Injury and CK-18 Outcomes From Linear Mixed Models
eFigure 3. Sensitivity Analyses of Individual Liver Enzymes (ALT, GGT, and AST) and Stratified Analyses by Child Sex Using BWQS
eFigure 4. Sensitivity Analyses for Shorter Half-life Chemicals (Phenols, Parabens, Phthalates and OP Pesticides) Stratifying Between Those Cohorts Who Measured Exposures in the Second Pregnancy Trimester vs Third Trimester
eFigure 5. Schematic Diagram of the Statistical Analysis Plan, Detailing the Sequences of the Analyses
eMethods. Description of HELIX Subcohort and Inclusion Criteria, Liver Outcome Assessment, Details on Statistical Methods, and Postnatal EDC Exposure Assessment
eResults. GLMER Results for Individual Chemicals and Results of Sensitivity Analyses
eTable 1. Prenatal EDC Exposure Assessment
eTable 2. Biological Matrices, Timing of Sample Collection, Laboratories, Analytical Methods, and Quality Controls for Prenatal EDC Exposures Assessment
eTable 3. Statistical Approaches for Analysis of Multiple Correlated Exposures
eTable 4. Log (Base = 2) Transformed Prenatal Blood and Serum Mean (SE) Concentrations of the Chemical Exposures
eTable 5. BWQS Models for Liver Injury and CK-18
eTable 6. Estimated Posterior Weights of Exposure Mixture Groups on Liver Injury and CK-18 Outcomes Using BWQS Models
eTable 7. BKMR Models for Liver Injury and CK-18
eTable 8. Estimated Posterior Inclusion Probabilities (PIPs) of Exposures in the Mixture Groups for Liver Injury and CK-18 Outcomes Using BKMR Models
eTable 9. Linear Mixed Effects Regression (LMER) Models for Individual Chemicals on Binary Liver Injury and Continuous CK-18 Outcomes
eTable 10. Analyses of Individual Liver Enzymes (ALT, AST, and GGT) Using BWQS Models
eTable 11. Stratified BWQS Analyses of Liver Injury and CK-18 by Sex of the Children
eTable 12. BWQS Analyses of Liver Injury and CK-18 After Adjusting for Postnatal Chemical Exposures
eTable 13. BWQS Analyses of Liver Injury and CK-18 After Adjusting for Maternal Diet Quality (Consumption of Fish, Fruits, and Vegetables in Times per Week)
eTable 14. BWQS Analyses of Liver Injury and CK-18 After Adjusting for Child BMI *z* Score
eReferences

© 2022 Midya V et al. *JAMA Network Open*.

This supplementary material has been provided by the authors to give readers additional information about their work.

© 2022 Midya V et al. *JAMA Network Open*.

eFigure 1. Directed Acyclic Graphs (DAGs) Elucidating Exposure-Outcome Association

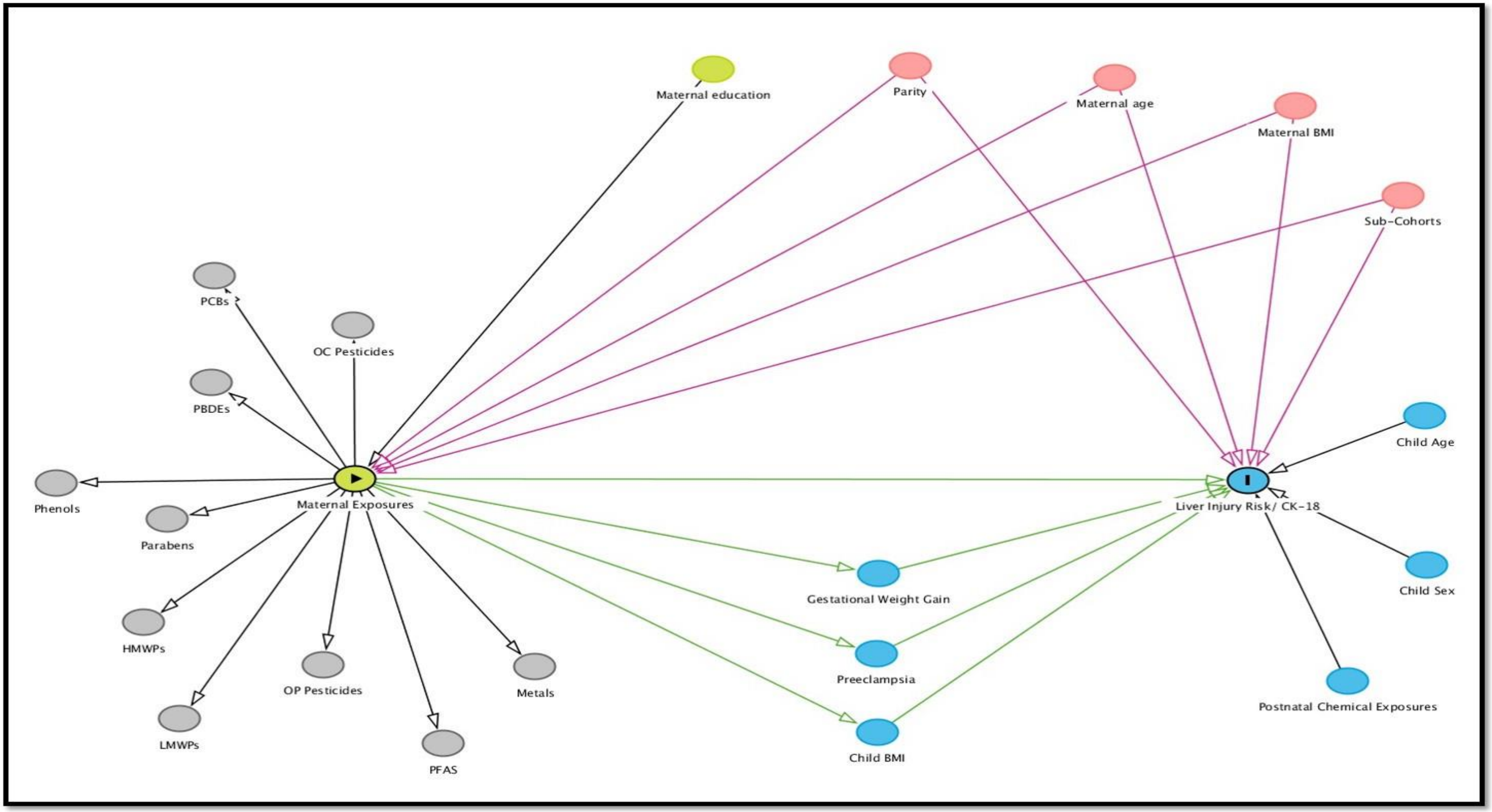

© 2022 Midya V et al. *JAMA Network Open*.

eFigure 2. Forest Plots for Individual Chemical Exposures for Liver Injury and CK-18 Outcomes From Linear Mixed Models

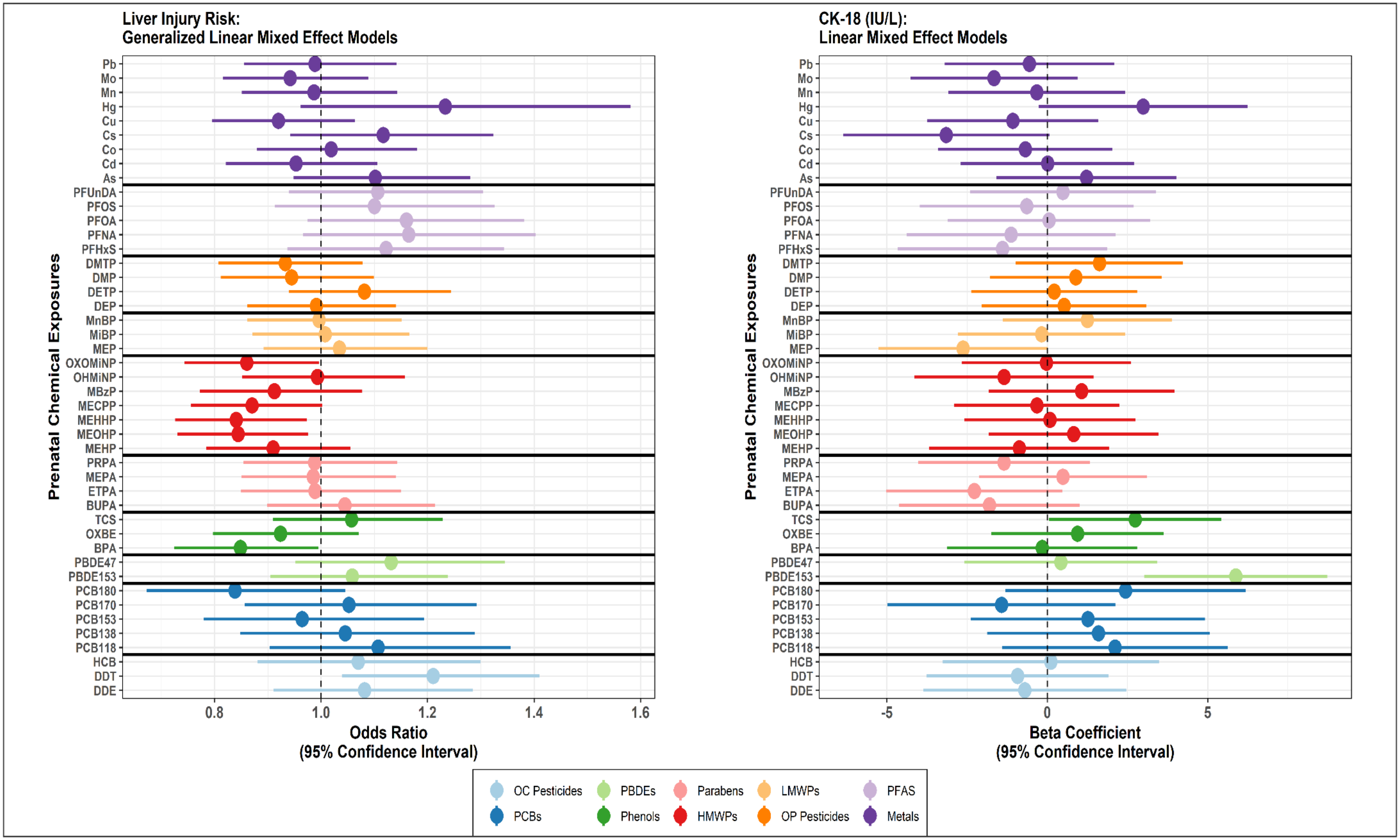

© 2022 Midya V et al. *JAMA Network Open*.

Odds ratio and 95% CI for binary outcome liver injury risk (A) and CK-18 (B) across all individual chemicals. The dots represent the point estimates (Betas) for a quartile increase in each exposure and corresponding horizontal lines denote the 95% CIs. Dotted black vertical lines represent the null association. All models were adjusted for sub-cohort, maternal age, maternal pre-pregnancy BMI, maternal education level parity, child sex and child age.

© 2022 Midya V et al. *JAMA Network Open*.

eFigure 3. Sensitivity Analyses of Individual Liver Enzymes (ALT, GGT, and AST) and Stratified Analyses by Child Sex Using BWQS

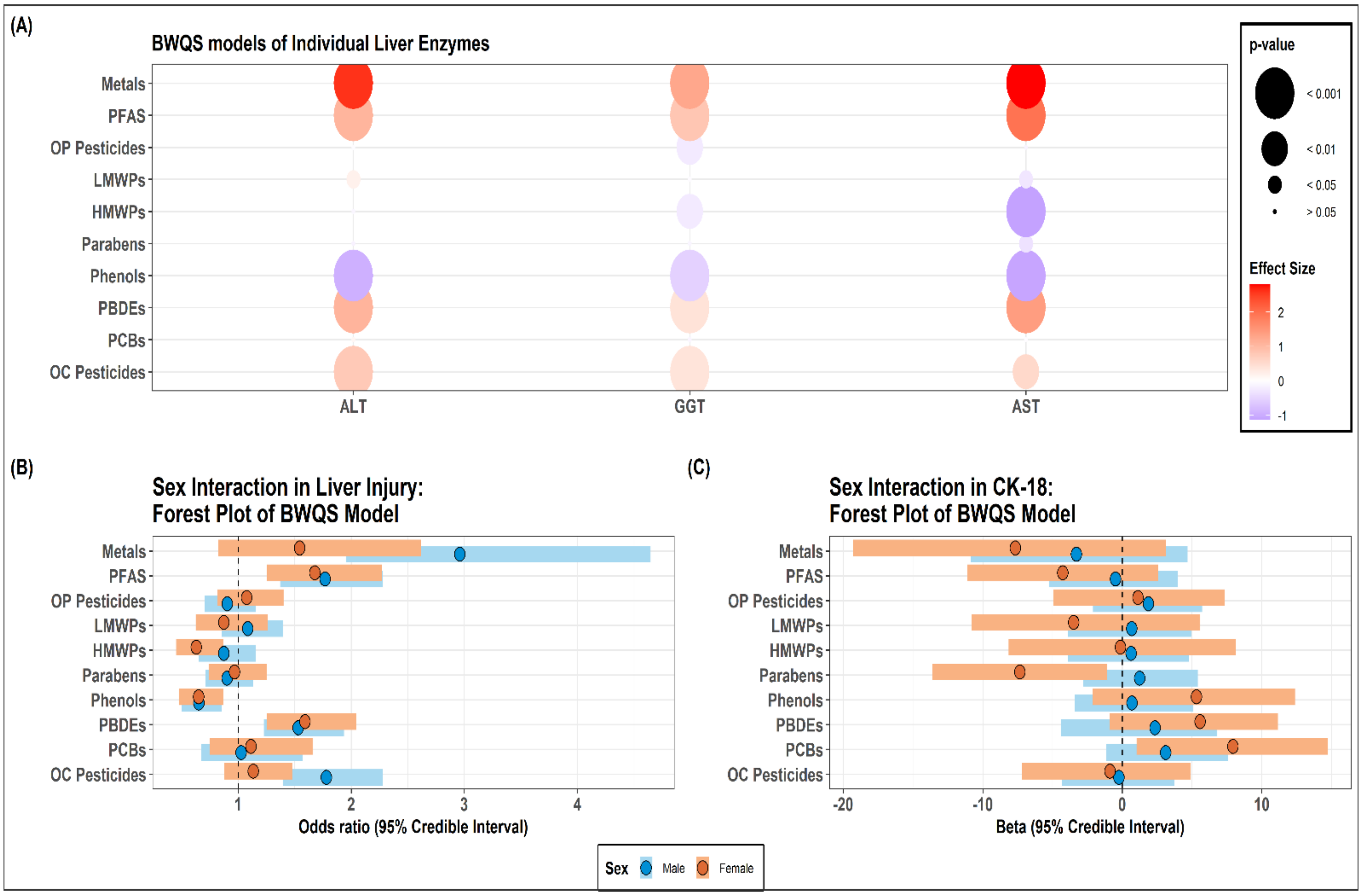

(A) Bubble plot for ALT, GGT and AST depicting the estimated Betas (through color gradient) and statistical significance of the associations through the size of the dots. Red and violet colors denote higher and lower effect sizes respectively. Effect sizes closer to zero are dimmed to white. Forest plot of BWQS models after stratifying by child sex in (B) Liver Injury and (C) CK-18. The dots in (B) and (C) indicate beta estimate and the bars around the dots imply 95% Credible Intervals. The dotted vertical line denotes null association. All models in (A) were adjusted for sub-cohort, maternal age, maternal pre-pregnancy BMI, maternal education level parity, child sex and child age.

© 2022 Midya V et al. *JAMA Network Open*.

eFigure 4. Sensitivity Analyses for Shorter Half-life Chemicals (Phenols, Parabens, Phthalates and OP Pesticides) Stratifying Between Those Cohorts Who Measured Exposures in the Second Pregnancy Trimester vs Third Trimester

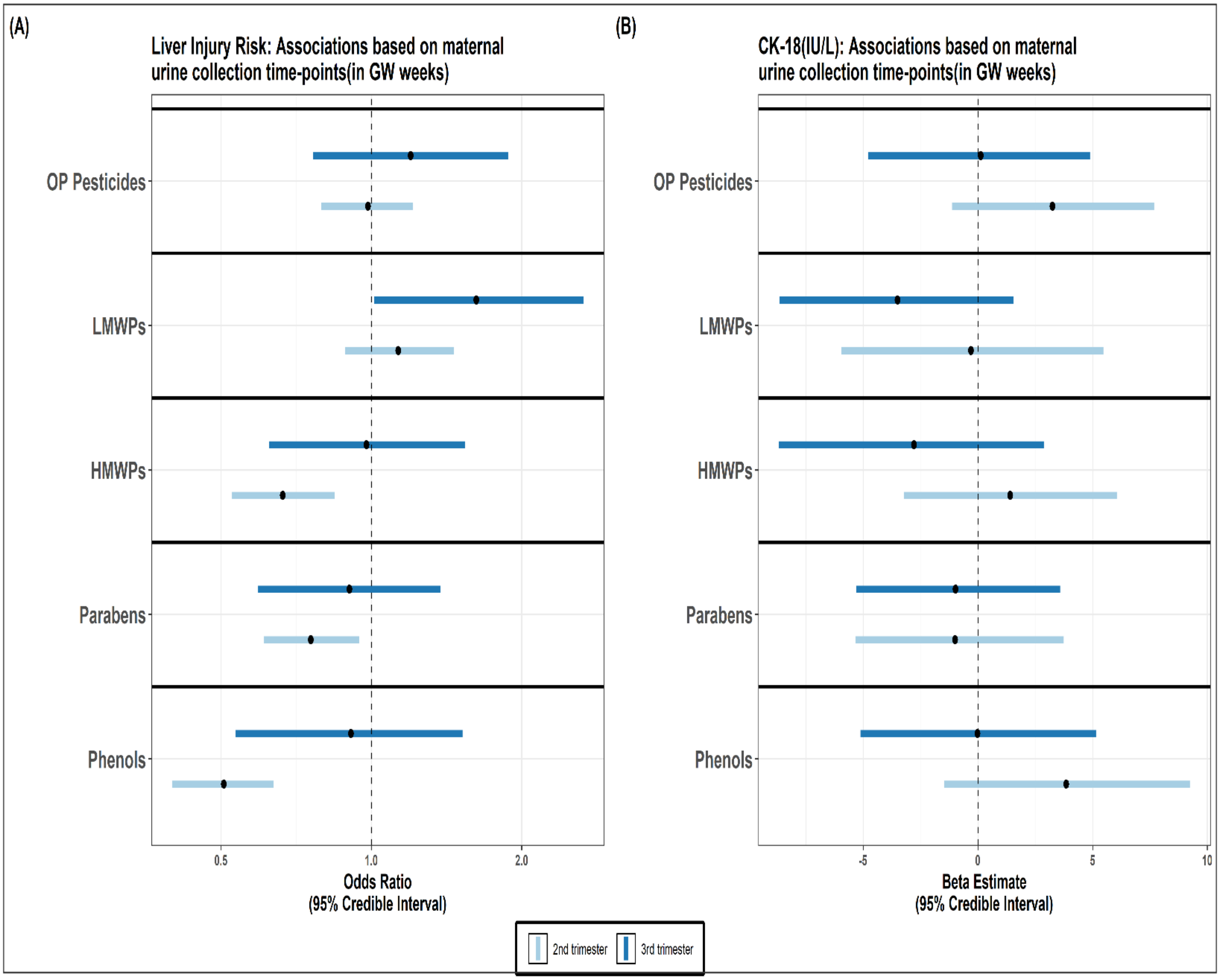

The dots in (A) and (B) indicate Odds ratio or beta estimate and the bars around the dots imply 95% Credible Intervals. The dotted vertical line denotes null association. All models were adjusted for sub-cohort, maternal age, maternal pre-pregnancy BMI, maternal education level parity, child sex and child age.

© 2022 Midya V et al. *JAMA Network Open*.

eFigure 5. Schematic Diagram of the Statistical Analysis Plan, Detailing the Sequences of the Analyses

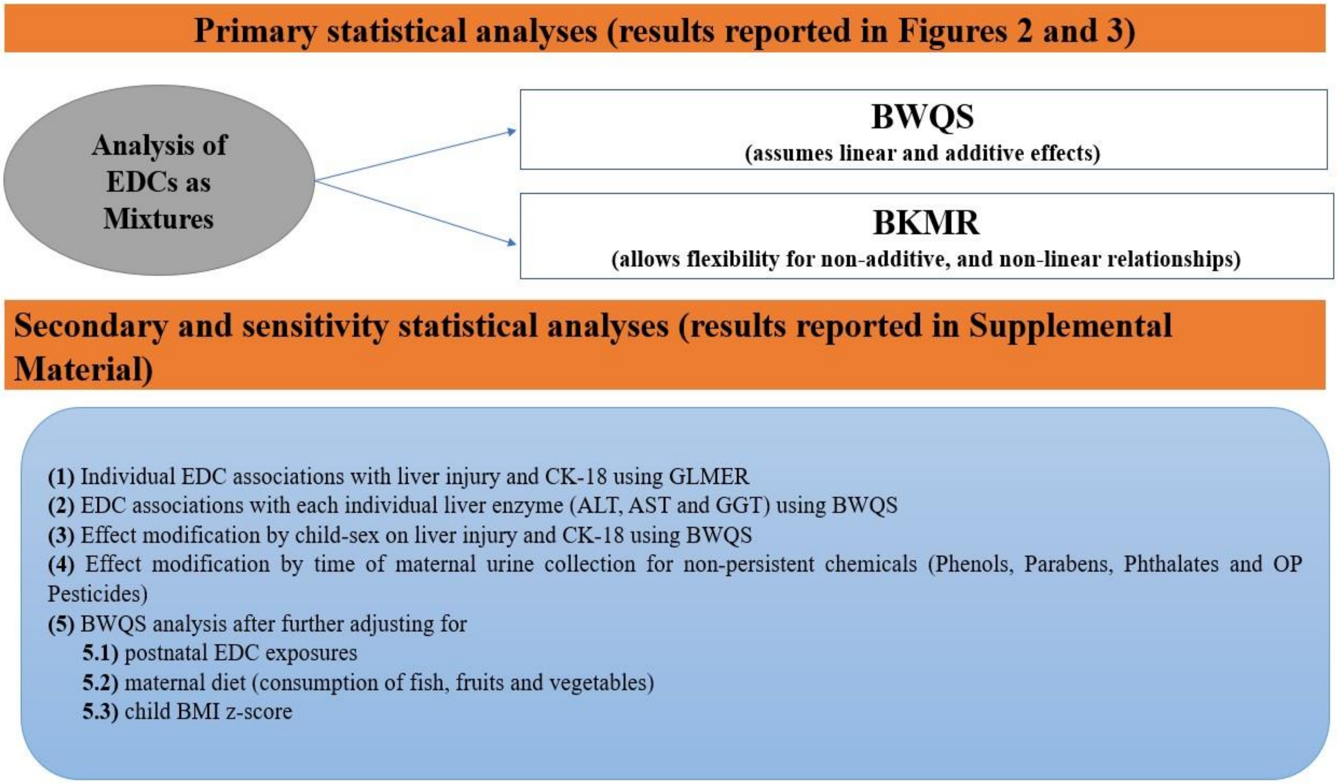

© 2022 Midya V et al. *JAMA Network Open*.

eMethods. Description of HELIX Subcohort and Inclusion Criteria, Liver Outcome Assessment, Details on Statistical Methods, and Postnatal EDC Exposure Assessment

The description of the HELIX sub-cohort and the eligibility criteria for inclusion has been obtained from Maitre et al., 2018 BMJ Open[1] and presented here verbatim.

HELIX sub-cohort:

"From the entire cohort, a sub-cohort of mother-child pairs was selected to be fully characterized for a broad suite of environmental exposures and 'omics' data, to be clinically examined and to have biological samples collected. A new follow-up visit was organized for these mother-child pairs between December 2013 and February 2016. Sub-cohort subjects were recruited from within the entire cohorts such that there were approximately 200 mother-child pairs from each of the six cohorts. Sub-cohort recruitment in the EDEN cohort was restricted to the Poitiers area and in the INMA cohort to the city of Sabadell."

"**Eligibility criteria for inclusion in the sub-cohort was** (a) age 6–11 years at the time of the visit, with a preference for ages 7–9 years if possible; (b) sufficient stored pregnancy blood and urine samples available for analysis of prenatal exposure biomarkers; (c) complete address history available from first to last follow-up point; (d) no serious health problems that may affect the performance of the clinical testing or impact the volunteer's safety (e.g., acute respiratory infection). In addition, the selection considered whether data on important covariates (diet, socioeconomic factors) were available. Each cohort selected participants at random from the eligible pool in the entire cohort and invited them to participate in this sub-cohort until the required number of participants was reached. In total, 1301 mother-child pairs with complete questionnaire and clinical examination data, and urine and blood samples, were included in the HELIX sub-cohort."

Liver Outcome Assessment

ALT, AST, and GGT in serum were measured at the Biochemistry Laboratory of the Clínica Universidad de Navarra, using homogenous enzymatic colorimetric methods on a Colorimetry Cobas 8000 analyzer (as described in the manufacturer's instructions (Roche Diagnostics GmbH, Mannheim, Germany)). CK-18 (a plasma caspase–generated fragment and novel marker of hepatocyte apoptosis, in children [2]) in serum was measured using ELISA (M30 Apoptosense® ELISA, PEVIVA).

© 2022 Midya V et al. *JAMA Network Open*.

Statistical Analyses

We used triangulation of available state-of-the-art statistical methods for exposure mixture analysis in environmental health studies to confirm the robustness of associations independently of the statistical approach used and identify consistent findings across all methods, as each method presents unique strengths and limitations. Firstly, to model group mixture associations under the assumption of linearity and additivity, Bayesian Weighted Quantile Sum (BWQS) regressions [3] were fitted where all chemicals belonging to a particular group were modelled at once. This method is useful when there is no prior information about the direction of the mixture-outcome association. BWQS does not select a priori the directionality of the association, rather utilizes a Dirichlet prior which is naturally incorporated in this Bayesian analysis. Further, the estimated group mixture coefficient is then mapped to the corresponding contributions of each of the individual exposures. These contributions or weights identify the relative importance of each individual exposure within the group mixture association. Note that, in each chemical group, the estimated weights sum up to one, and are constrained to be positive.

Secondly, after relaxing the assumptions of linearity and additivity in group mixture models, Bayesian Kernel Machine Regression (BKMR) [4] was used to identify more flexible non-linear group associations. This tool utilizes the Kernel machine regression framework (also called Gaussian process regression) to flexibly model the exposure-outcome association without assuming any stringent structure. BKMR is a blend of usual machine learning framework and classical statistics, which allows to flexibly model the exposure-response function after controlling for the effect of the confounders. To control for multi-collinearity among the exposures and variable selection, it uses the so-called "Spike and Slab" prior. Since, it doesn't assume any structure for the exposure-response function, BKMR doesn't provide an overall estimate for group association. But similar to BWQS, the individual contribution to the exposure-response curve can be estimated using the Posterior Inclusion Probabilities (PIPs). Note that, in each chemical group, the estimated PIPs were scaled to sum up to one, to denote the relative inclusion of each chemical exposure to the overall group mixture association. This facilitates direct comparison with weights derived from BWQS.

Thirdly, we used Generalized Linear Mixed Effect Regression (GLMER) models to evaluate the associations of each individual chemical with the liver outcomes while avoiding multi-collinearity. All models were adjusted for confounders. For ease of interpretation and comparison of estimates between the methods, all the prenatal chemical exposures (which were log transformed before) were converted to quartiles. See eTable 3 for a summary of advantages and limitations of using each one of the three statistical approaches. BWQS was used as the main model for its simplicity while interpreting the results and computational speed.

© 2022 Midya V et al. *JAMA Network Open*.

A single imputation of imputed biomarker concentrations below the limit of detection was conducted using a quantile regression approach for the imputation of left-censored missing data. The urinary biomarker concentrations were divided by creatinine concentration and the haemal lipophilic biomarker concentrations were standardized and expressed in ng/g of total lipids in serum or plasma. Further, the missing data for exposure biomarker concentrations were imputed for 100 imputed datasets via a chained equations algorithm. See Appendix Table 5 of [5] for more details.

Postnatal EDC exposure assessment for Sensitivity Analysis

Postnatal EDCs exposures were measured in blood and urine samples of children collected during the HELIX sub-cohort follow-up visit at ages 6-11 years. Blood draws were performed after a median (5th, 95th percentile) fasting time of 3.3 (2.2, 5.9) hours. All blood samples were collected and processed after following identical predefined standardized protocols for all the six sub-cohorts. To provide slightly long-term exposure assessment than could be achieved with one spot urine sample, a combination of urine samples from the night before the visit and from the first morning void on the day of the visit was used [6]. See [1, 6, 7] for more details on postnatal EDC exposure measurements.

eResults. GLMER Results for Individual Chemicals and Results of Sensitivity Analyses

GLMER results

Analysis of individual chemicals using GLMER indicated DDT was associated with increased odds of liver injury (OR[95%CI] by one quartile increase=1.21[1.04, 1.41]) (eFigure 2A). The other OC pesticides (DDE and HCB) and PBDEs were positively associated with increased odds of liver injury risk but associations did not reach statistical significance. BPA and certain HMWPs, including MECPP, MEHHP and MEOHP were associated with decreased odds of liver injury (0.85[0.72, 0.99], 0.87[0.76, 1.00], 0.84[0.73, 0.97], 0.84[0.73, 0.98] respectively) (eFigure 2A). Both the PBDEs were positively associated with CK-18, and in particular a quartile increase of PBDE153 was significantly associated with increase in CK-18 (Beta [95%CI] =5.88 U/L [3.03, 8.74]) (eFigure 2B). Most PCBs tended to be positively associated with CK-18 but the associations were not statistically significant. One of the phenols, triclosan (TCS) was also positively associated with CK-18 (Beta [95%CI] =2.74 U/L [0.05, 5.43]) (eFigure 4B). All the estimated coefficients and 95% CIs from the mixed models are presented in eFigure 2 (and eTable 9). Multiple comparison error was controlled by calculating q-values.

© 2022 Midya V et al. *JAMA Network Open*.

SENSITIVITY ANALYSES

EFFECT MODIFICATION BY SEX

We tested for effect modification and stratified analyses by sex (eFigure 3 and eTable 11). We observed differences in the effect sizes for liver injury by sex for OC pesticides and metals with stronger associations suggested in males compared to females (OC pesticides: OR[95% CrI] by quartile increase in the OC mixture = 1.78 [1.40, 2.28] in males versus 1.13 [0.88, 1.48] in females; and metals: OR[95% CrI] by quartile increase in Metal mixture = 2.96 [1.95, 4.65] in males versus 1.54 [0.83, 2.62] in females). There was no significant sex interaction between any of the EDC mixtures and CK-18.

ROBUSTNESS OF ASSOCIATIONS AFTER CONTROLING FOR POSTNATAL EDC EXPOSURES, MATERNAL DIET IN PREGNANCY, TIMING OF URINE SAMPLE COLLECTION FOR NON-PERSISTENT CHEMICALS AND CHILD BMI Z-SCORE

The strength and directionality of all EDC associations with liver injury remained unchanged after adjusting the statistical models for EDC exposures measured in child blood or urine at age ~8 years (eTable 12) and maternal diet (consumption of fish, fruits and vegetables; in times per week) (eTable 13). For the associations with CK-18, the PCBs effect estimate was slightly attenuated and did not remain statistically significant after adjustment for post-natal PCB exposures (eTable 12). Adjusting models for child BMI z-scores did not meaningfully change effect estimates for any of the associations of interest (<10% change in estimates) (eTable 14). Further, sensitivity analysis was also conducted for shorter half-life chemicals stratifying analyses between those cohorts who measured non-persistent EDCs in urine in the second pregnancy trimester versus INMA (that collected urine around 34 (SD:1.3) weeks of gestation) and we did not find any significant differences in the effect estimates (eFigure 4). Overall, we did not observe meaningful changes in effect estimates after controlling for postnatal EDC exposures, maternal diet or child BMI in pregnancy in statistical models.

© 2022 Midya V et al. *JAMA Network Open*.

eTable 1. Prenatal EDC Exposure Assessment

Chemical exposure group	Individual chemicals	Biological matrix (Unit)	Limit of Detections (LOD) used in NIPH[1]
Organochlorine Pesticides (OC Pesticides)	DDE, DDT, HCB	serum or plasma (ng/g of lipids)	0.61 pg/g, 0.3 pg/g, 1.52 pg/g
Polychlorinated Biphenyls (PCBs)	PCB congeners 118, 138, 153, 170, 180	serum or plasma (ng/g of lipids)	0.3 pg/g, 0.61 pg/g, 0.61 pg/g, 0.61 pg/g, 0.91 pg/g
Polybrominated diphenyl ethers (PBDEs)	PBDE47, PBDE153	serum or plasma (ng/g of lipids)	0.15 pg/g, 0.3 pg/g
Parabens	MEPA, ETPA, PRPA, BUPA	Urine (µg/g of creatinine)	0.03 µg/L, 0.03 µg/L, 0.03 µg/L, 0.06 µg/L,
Phenols	OXBE, TCS, BPA	Urine (µg/g of creatinine)	0.03 µg/L, 0.03 µg/L, 0.03 µg/L
High Molecular Weight Phthalates (HMWPs)	MBzP, MECPP, MEHP, MEHHP, MEOHP, OHMiNP, OXOMiNP	Urine (µg/g of creatinine)	0.06 µg/L, 0.61 µg/L, 0.15 µg/L, 0.12 µg/L, 0.15 µg/L, 0.06 µg/L, 0.06 µg/L
Low Molecular Weight Phthalates (LMWPs)	MEP, MiBP, MnBP,	Urine (µg/g of creatinine)	0.15 µg/L, 0.15 µg/L, 0.15 µg/L

© 2022 Midya V et al. *JAMA Network Open*.

Organophosphate Pesticides (OP Pesticides)	DMP, DMTP, DEP, DETP	Urine (µg/g of creatinine)	0.36 µg/L, 0.06 µg/L, 0.09 µg/L, 0.13 µg/L,
Per- and polyfluoroalkyl substances (PFAS)	PFHxS, PFNA, PFOA, PFOS, PFUnDA	serum or plasma or whole blood (µg/L)	0.02 µg/L, 0.02 µg/L, 0.02 µg/L, 0.02 µg/L, 0.02 µg/L
Metals	As, Cd, Cs, Co, Cu, Pd, Mn, Hg, Mo	whole blood or cord blood (µg/L)	0.3 µg/L, 0.003 µg/L, 0.02 µg/L, 0.02 µg/L, 0.3 µg/L, 0.15 µg/L, 0.15 µg/L, 0.02 µg/L, 0.06 µg/L

Note: ***As***, Inorganic arsenic; ***BPA***, Bisphenol A; ***BUPA***, N-butyl paraben; ***Cd***, Cadmium; ***Cs***, Caesium; ***Co***, Cobalt; ***Cu,*** Copper; ***DDE***, 4,4′dichlorodiphenyl dichloroethylene; ***DDT***, 4,4′dichlorodiphenyltrichloroethane; ***DEP***, diethyl phosphare; ***DEHP***, di(2-ethylhexyl) phthalate; ***DETP***, diethyl thiophosphate; ***DEDTP***, diethyl dithiophosphate; ***DMP***, dimethyl phosphate; ***DMTP***, dimethyl thiophosphate; ***DMDTP***, dimethyl dithiophosphate; ***ETPA***, ethyl paraben; ***HCB***, hexachlorobenzene; ***Hg***, Mercury; ***MBzP***, mono benzyl phthalate; ***MECPP***, mono-2-ethyl 5-carboxypentyl phthalate; ***MEHP***, mono(2-ethylhexyl) phthalate; ***MEHHP***, mono(2-ethyl-5-hydroxyhexyl) phthalate; ***MEOHP***, mono(2-ethyl-5-oxohexyl) phthalate; ***MEP***, monoethyl phthalate; ***MEPA***, methyl paraben; ***MiBP***, mono-iso-butyl phthalate; ***Mn***, Manganese; ***MnBP***, mono-n-butyl phthalate; ***Mo***, Molybdenum; ***OHMiNP***, mono-4-methyl-7-hydroxyoctyl phthalate; ***OXBE***, oxybenzone; ***OXOMiNP***, mono-4-methyl-7-oxooctyl phthalate; ***Pb***, Lead; ***PBDE47***, 2,2′,4,4′-tetra-bromodiphenyl ether; ***PBDE153***, 2,2′,4,4′,5,5′-hexa-bromodiphenyl ether; ***PCBs***, polychlorinated biphenyl –118, 138, 153, 170, 180; ***PFHxS***, Perfluorohexane sulfonate; ***PFNA***, perfluorononanoate; ***PFOA***, perfluorooctanoate; ***PFOS***, perfluorooctane sulfonate; ***PFUnDA***, perfluoroundecanoate; ***PRPA,*** propyl paraben; ***TCS***, triclosan;

[1] NIPH : Norwegian Institute of Public Health; more details on the limit of quantification (LOQ) and limit of detection (LOD) for each EDC exposure analyzed by NIPH or other associated laboratories are described in SI, Excel Tables S5 of [6].

© 2022 Midya V et al. *JAMA Network Open*.

eTable 2. Biological Matrices, Timing of Sample Collection, Laboratories, Analytical Methods, and Quality Controls for Prenatal EDC Exposures Assessment

Cohort	**BiB**	**EDEN**	**KANC**	**INMA**	**RHEA**	**MOBA**
OC Pesticides and PCBs						
Biological matrix	Serum/plasma	Serum	-	Serum	Serum	Plasma
Collection time-point maternal samples, gestational week (mean, (SD))	26.6 (1.4)	26.1 (1.2)	39.4 (1.3)	13.7 (2.0)	14.1 (3.7)	18.7 (0.9)
Laboratory	NIPH (Norway)	NIPH (Norway)	NIPH (Norway)	LSPG (Spain)	National Institute for Health and Welfare, Chemical Exposure Unit, Kuopio (Finland)	NIPH (Norway)
Analytical method	GC-MS/MS	GC-MS/MS	GC-MS/MS	GC-MS	GC-MS/MS	GC-MS/MS
Interlaboratory comparison	Participated 3 times in the AMAP inter-laboratory comparison study (Arctic Monitoring and Assessment Program “Ring Test for Persistent Organic Pollutants in Human Serum) during the period when HELIX samples were analyzed. Each round included 3 samples. The samples are spiked, and most analytes are in a much higher concentration range than our samples usually are. Despite these factors Z- scores below 2 were obtained for all contaminants except for PCB 153, and for PCB 170 in one sample.					
Standard reference material	Two different SRMs were obtained from NIST (National Institute of Standards and Technology, USA), Organic Contaminants in Human Serum, non-fortified (1957) and fortified (1958). The RSDs of the 6 different injections of the non-fortified sample (1957) were 2-18%. The deviation from the certified values was 2-20%, except for HCB (50%) and PCB-153 (76%), where a small blank contribution may be the problem. The RSDs of the 6 different injections of the fortified sample (1958) were 2-20%, and for most analytes around 5%. The deviation from the certified values were 2-17%, except for DDT (-40%).					

© 2022 Midya V et al. *JAMA Network Open*.

Reference for the analytic method	Modified (Caspersen et al. 2016)	Modified (Caspersen et al. 2016)	Modified (Caspersen et al. 2016)	(Goñi et al. 2007)	(Koponen et al. 2013)	Modified (Caspersen et al. 2016)
PBDEs						
Cohort	**BiB**	**EDEN**	**KANC**	**INMA**	**RHEA**	**MOBA**
Biological matrix	Serum/plasma	Serum	-	Serum	Serum	Plasma
Collection time-point maternal samples, gestational week (mean, (SD))	26.6 (1.4)	26.1 (1.2)	39.4 (1.3)	13.7 (2.0)	14.1 (3.7)	18.7 (0.9)
Laboratory	NIPH (Norway)	NIPH (Norway)	NIPH (Norway)	NIPH (Norway)	National Institute for Health and Welfare, Chemical Exposure Unit, Kuopio (Finland)	NIPH (Norway)
Analytical method	GC-MS/MS	GC-MS/MS	GC-MS/MS	GC-MS	GC-MS/MS	GC-MS/MS
Interlaboratory comparison	We participated 3 times in the AMAP interlaboratory comparison study (Arctic Monitoring and Assessment Program “Ring Test for Persistent Organic Pollutants in Human Serum) during the period when HELIX samples were analysed. Each round included 3 samples. The samples are spiked, and most analytes are in a much higher concentration range than our samples usually are. Despite these factors Z- scores below 2 were obtained for all contaminants.					
Standard reference material	Two different SRMs were obtained from NIST (National Institute of Standards and Technology, USA), Organic Contaminants in Human Serum, non-fortified (1957) and fortified (1958). The RSDs of the 6 different injections of the non-fortified sample (1957) were 2-18%. The deviation from the certified values was 2-20%. The RSDs of the 6 different injections of the fortified sample (1958) were 2-20%, and for most analytes around 5%. The deviation from the certified values were 2-17%.					

© 2022 Midya V et al. *JAMA Network Open*.

Reference for the analytic method	Modified (Caspersen et al. 2016)	Modified (Caspersen et al. 2016)	Modified (Caspersen et al. 2016)	Modified (Caspersen et al. 2016)	(Koponen et al. 2013)	Modified (Caspersen et al. 2016)
PFAS						
Cohort	**BiB**	**EDEN**	**KANC**	**INMA**	**RHEA**	**MOBA**
Biological matrix	Serum/plasma	Serum	Whole blood	Plasma	Serum	Plasma
Collection time-point maternal samples, gestational week (mean, (SD))	26.6 (1.4)	26.1 (1.2)	39.4 (1.3)	13.7 (2.0)	14.1 (3.7)	18.7 (0.9)
Laboratory	NIPH (Norway)	NIPH (Norway)	NIPH (Norway)	Institute for Occupational Medicine, RWTH Aachen University (Germany)	NIPH (Norway)	NIPH (Norway)
Analytical method	GC-MS	GC-MS	GC-MS	Online column-switching LC–MS–MS analysis	GC-MS	GC-MS
Interlaboratory comparison	We participated 3 times in the AMAP interlaboratory comparison study (Arctic Monitoring and Assessment Program “Ring Test for Persistent Organic Pollutants in Human Serum) during the period where HELIX samples were analysed. Each round included 3 samples. The samples are spiked in a wide concentration range. Z- scores below 2 were obtained for all contaminants except for PFUnDA in two samples.					

© 2022 Midya V et al. *JAMA Network Open*.

Standard reference material	In total 15 samples from 4 rounds of the AMAP interlaboratory comparison study (Arctic Monitoring and Assessment Program "Ring Test for Persistent Organic Pollutants in Human Serum) were analysed during the period where HELIX samples were analysed, and the mean deviation from the assigned value varied between 8 and 17%.					
Reference for the analytic method	(Haug et al. 2009)	(Haug et al. 2009)	(Poothong et al. 2017)	(Manzano-Salgado et al. 2015)	(Haug et al. 2009)	(Haug et al. 2009)
Metals and semi-metals						
Cohort	**BiB**	**EDEN**	**KANC**	**INMA**	**RHEA**	**MOBA**
Biological matrix	Whole blood	Whole blood	Whole blood	whole blood (Hg was measured in Cord blood)	Whole blood	Whole blood
Timing of sample collection, mean GW (SD)	26.6 (1.4)	26.1 (1.2)	39.4 (1.3)	13.7 (2.0)	14.1 (3.7)	18.7 (0.9)
Laboratory	ALS Scandinavia (Sweden)	ALS Scandinavia (Sweden)	ALS Scandinavia (Sweden)	Hg; LSPA (Spain)	ALS Scandinavia (Sweden)	ALS Scandinavia (Sweden)
Analytical method	ICP-SFMS	ICP-SFMS	ICP-SFMS	AAS	ICP-SFMS	ICP-SFMS
Standard reference material	In total 15 samples of two Seronorm whole blood reference materials were provided to ALS Scandinavia which performed analyses of toxic and essential elements of the HELIX samples. The samples were blinded for the laboratory. The mean deviation from the assigned value varied between 1 and 17%, except for Co which was 53%.					
Reference for the analytic method	(Rodushkin et al. 2000)	(Rodushkin et al. 2000)	(Rodushkin et al. 2000)	(Ramon et al. 2011)	(Rodushkin et al. 2000)	(Rodushkin et al. 2000)

© 2022 Midya V et al. *JAMA Network Open*.

Phthalate metabolites						
Cohort	**BiB**	**EDEN**	**KANC**	**INMA**	**RHEA**	**MOBA**
Biological matrix	Urine	Urine	-	Urine	Urine	Urine
Timing of sample collection, mean GW (SD)	26.6 (1.4)	26.1 (1.2)	NA	34.2 (1.3)	14.1 (3.7)	18.7 (0.9)
Laboratory	NIPH (Norway)	NIPH (Norway)	NIPH (Norway)	Bioanalysis Research Group at the Hospital del Mar Medical Research Institute (Spain)	NIPH (Norway)	NIPH (Norway)
Analytical method	LC-MS/MS	LC-MS/MS	LC-MS/MS	HPLC-MS	LC-MS/MS	LC-MS/MS
Standard reference material	In total 42 samples of NIST reference material SRM 3673 were analysed during the period where HELIX samples were analysed, and the mean deviation from the assigned value varied between 1 and 25%.					
Reference for the analytic method	(Sabaredzovic et al. 2015)	(Sabaredzovic et al. 2015)	(Sabaredzovic et al. 2015)	(Valvi et al. 2015)	(Sabaredzovic et al. 2015)	(Sabaredzovic et al. 2015)
Phenols and Parabens						
Cohort	**BiB**	**EDEN**	**KANC**	**INMA**	**RHEA**	**MOBA**
Biological matrix	Urine	Urine	-	Urine	Urine	Urine
Timing of sample collection, mean GW (SD)	26.6 (1.4)	26.1 (1.2)	NA	34.2 (1.3)	14.1 (3.7)	18.7 (0.9)

© 2022 Midya V et al. *JAMA Network Open*.

Laboratory	NIPH (Norway)	National Center for Environmental Health laboratory at the CDC (US)	NIPH (Norway)	NIPH (Norway)	NIPH (Norway)	NIPH (Norway)
Analytical method	UPLC-MS/MS	Described in (Philippat et al. 2012)	UPLC-MS/MS	UPLC-MS/MS	UPLC-MS/MS	UPLC-MS/MS
Interlaboratory comparison	We participated twice in the External Quality Assessment Scheme for organic substances in urine – OSEQAS, for BPA and TCS. Each round included 2 samples. Z- scores below 2 were obtained for both contaminants in all samples.					
Standard reference material	In total 145 samples of NIST reference material SRM 3673 were analysed during the period where HELIX samples were analysed, and the mean deviation from the assigned value varied between 1 and 19%, except BPA which was 63%.					
Reference for the analytic method	(Sakhi et al. 2018)	(Philippat et al. 2012)	(Sakhi et al. 2018)	(Sakhi et al. 2018)	(Sakhi et al. 2018)	(Sakhi et al. 2018)
OP pesticides						
Cohort	**BiB**	**EDEN**	**KANC**	**INMA**	**RHEA**	**MOBA**
Biological matrix	Urine	Urine	-	Urine	Urine	Urine
Timing of sample collection, mean GW (SD)	26.6 (1.4)	26.1 (1.2)	NA	34.2 (1.3)	14.1 (3.7)	18.7 (0.9)
Laboratory	NIPH (Norway)	NIPH (Norway)	NIPH (Norway)	NIPH (Norway)	NIPH (Norway)	NIPH (Norway)
Analytical method	UPLC-TOF	UPLC-TOF	UPLC-TOF	UPLC-TOF	UPLC-TOF	UPLC-TOF
Reference	(Cequier et al. 2016)	(Cequier et al. 2016)	(Cequier et al. 2016)	(Cequier et al. 2016)	(Cequier et al. 2016)	(Cequier et al. 2016)

© 2022 Midya V et al. *JAMA Network Open*.

NA, not available; SD, standard deviation; BPA, bisphenol A; DDT, dichlorodiphenyltrichloroethane; HCB, hexachlorobenzene; OC, organochlorine compound; OP, organophosphate; PBDE, polybrominated diphenyl ether; PCB, polychlorinated biphenyl; PFAS, per- and polyfluoroalkyl substance; PFUnDA, perfluoroundecanoate; TCS, triclosan; RSD, relative standard deviation; SRM, Standard reference material; AAS, thermal decomposition, amalgamation and atomic absorption spectrometry; GC-MS/MS, gas chromatography coupled with tandem mass spectrometry; GC-MS, gas chromatography mass spectrometry; GC-MS-NICI, gas chromatography-negative-ion chemical ionization mass spectrometry; HPLC-MS, ultra-performance liquid chromatography coupled to tandem mass spectrometry; ICP-SFMS, conductively coupled plasma-sector field mass spectrometry; LC-MS/MS, liquid chromatography-mass spectrometry; Q-ICP-MS, inductively coupled plasma quadruple mass spectrometry; SPE-HPLC–MS/MS, on-line solid-phase extraction coupled to isotope dilution high performance liquid chromatography-tandem mass spectrometry; UPLC-MS/MS, ultra-performance liquid chromatography-tandem mass spectrometry; UPLC-TOF, ultra-performance liquid chromatography coupled to time-of-flight mass spectrometry; AMAP, Arctic Monitoring and Assessment Program "Ring Test for Persistent Organic Pollutants in Human Serum"; CDC, Centers for Disease Control and Prevention; LSPA, Laboratorio de Salud Pública de Alava; LSPG, Laboratorio de Salud Pública de Guipúzcoa; NIPH, Norwegian Institute of Public Health; NIST, National Institute of Standards and Technology, USA; BiB, Born in Bradford; EDEN, Étude des Déterminants Pré et Postnatals du Développement et de la Santé de l'Enfant; INMA, Infancia y Medio Ambiente; MOBA, the Norwegian Mother, Father and Child Cohort Study; KANC, Kaunas Cohort; RHEA, Mother-Child Cohort in Crete.

Adapted from [5, 6]

© 2022 Midya V et al. *JAMA Network Open*.

eTable 3. Statistical Approaches for Analysis of Multiple Correlated Exposures

Methods	Advantage	Limitations
Bayesian Weighted Quantile Sum (BWQS) Regression	• Estimates the mixture-outcome association and the contribution of each chemical to the mixture<br>• Direction of the association is estimated based on data	• Does not consider non-additive and non-linear relationships<br>• Possibility of overfitting due to lack of penalization
Bayesian Kernel Machine Regression (BKMR)	• Estimate the association between chemicals and the outcome allowing non-additive, and non-linear relationships	• Use of Spike and Slab prior on relatively smaller numbers of predictors might yield false negatives<br>• Might be over fitted due to higher flexibility
Generalized Linear Mixed Effect Regression (GLMER)	• Estimates linear associations between individual chemicals and the outcome<br>• Ease of interpretation and implementation	• Does not consider correlation among chemicals, thus fails to calculate robust standard errors and increases spurious findings

© 2022 Midya V et al. *JAMA Network Open*.

eTable 4. Log (Base = 2) Transformed Prenatal Blood and Serum Mean (SE) Concentrations of the Chemical Exposures

Chemical Group	Individual Chemical Exposures	Mean of Log (base = 2) transformed concentrations in IU/L	Standard Errors (SE)
OC Pesticides	DDE	6.59	0.03
	DDT	1.47	0.03
	HCB	3.96	0.02
PCBs	PCB118	2.16	0.02
	PCB138	4.06	0.02
	PCB153	4.95	0.02
	PCB170	2.81	0.03
	PCB180	4.18	0.03
PBDEs	PBDE153	-0.99	0.06
	PBDE47	0.07	0.04
Phenols	OXBE	3.43	0.08
	BPA	2.53	0.04
	TCS	3.96	0.08
Parabens	BUPA	1.92	0.07
	ETPA	3.38	0.07
	MEPA	7.77	0.06
	PRPA	5.88	0.06
HMWPs	MBzP	3.93	0.04
	MECPP	6.02	0.02
	MEHHP	5.04	0.03
	MEHP	3.80	0.03

© 2022 Midya V et al. *JAMA Network Open*.

	MEOHP	4.67	0.02
	OHMiNP	0.77	0.03
	OXOMiNP	0.88	0.03
LMWPs	MEP	8.20	0.04
	MiBP	6.08	0.02
	MnBP	5.77	0.03
OP Pesticides	DEP	2.57	0.03
	DETP	-0.12	0.06
	DMP	3.80	0.03
	DMTP	3.12	0.04
PFAS	PFHxS	-0.11	0.02
	PFNA	0.23	0.02
	PFOA	1.89	0.02
	PFOS	3.49	0.02
	PFUnDA	-1.72	0.02
Metals	As	0.62	0.04
	Cd	-1.33	0.02
	Co	-1.47	0.02
	Cs	1.56	0.011
	Cu	11.41	0.01
	Hg	1.70	0.03
	Mn	4.28	0.01

© 2022 Midya V et al. *JAMA Network Open*.

	Mo	0.18	0.01
	Pb	4.09	0.01

Note: ***As***, Inorganic arsenic; ***BPA***, Bisphenol A; ***BUPA***, N-butyl paraben; ***Cd***, Cadmium; ***Cs***, Caesium; ***Co***, Cobalt; ***Cu,*** Copper; ***DDE***, 4,4′dichlorodiphenyl dichloroethylene; ***DDT***, 4,4′dichlorodiphenyltrichloroethane; ***DEP***, diethyl phosphare; ***DEHP***, di(2-ethylhexyl) phthalate; ***DETP***, diethyl thiophosphate; ***DEDTP***, diethyl dithiophosphate; ***DMP***, dimethyl phosphate; ***DMTP***, dimethyl thiophosphate; ***DMDTP***, dimethyl dithiophosphate; ***ETPA***, ethyl paraben; ***HCB***, hexachlorobenzene; ***Hg***, Mercury; ***MBzP***, mono benzyl phthalate; ***MECPP***, mono-2-ethyl 5-carboxypentyl phthalate; ***MEHP***, mono(2-ethylhexyl) phthalate; ***MEHHP***, mono(2-ethyl-5-hydroxyhexyl) phthalate; ***MEOHP***, mono(2-ethyl-5-oxohexyl) phthalate; ***MEP***, monoethyl phthalate; ***MEPA***, methyl paraben; ***MiBP***, mono-iso-butyl phthalate; ***Mn***, Manganese; ***MnBP***, mono-n-butyl phthalate; ***Mo***, Molybdenum; ***OHMiNP***, mono-4-methyl-7-hydroxyoctyl phthalate; ***OXBE***, oxybenzone; ***OXOMiNP***, mono-4-methyl-7-oxooctyl phthalate; ***Pb***, Lead; ***PBDE47***, 2,2′,4,4′-tetra-bromodiphenyl ether; ***PBDE153***, 2,2′,4,4′,5,5′-hexa-bromodiphenyl ether; ***PCBs***, polychlorinated biphenyl –118, 138, 153, 170, 180; ***PFHxS***, Perfluorohexane sulfonate; ***PFNA***, perfluorononanoate; ***PFOA***, perfluorooctanoate; ***PFOS***, perfluorooctane sulfonate; ***PFUnDA***, perfluoroundecanoate; ***PRPA,*** propyl paraben; ***TCS***, triclosan;

© 2022 Midya V et al. *JAMA Network Open*.

eTable 5. BWQS Models for Liver Injury and CK-18

	Liver Injury Risk		CK-18	
	**OR**	**95% CrI**	**Beta**	**95% CrI**
**OC Pesticides**	1.44	(1.21, 1.71)	-0.56	(-4.05, 2.89)
**PCBs**	1.05	(0.78, 1.42)	5.84	(1.69, 10.08)
**PBDEs**	1.57	(1.34, 1.84)	6.46	(3.09, 9.92)
**Phenols**	0.66	(0.54, 0.78)	2.59	(-1.20, 6.49)
**Parabens**	0.92	(0.77, 1.11)	-2.60	(-6.23, 1.16)
**HMWPs**	0.74	(0.60, 0.91)	0.07	(-4.40, 4.27)
**LMWPs**	1.02	(0.84, 1.25)	-1.71	(-6.29, 3.74)
**OP Pesticides**	0.98	(0.82, 1.18)	1.69	(-1.90, 4.98)
**PFAS**	1.73	(1.45, 2.09)	-1.99	(-6.19, 1.87)
**Metals**	2.21	(1.65, 3.02)	-6.28	(-13.14, 0.60)

The estimates are presented as ***(OR (95% Credible Interval) or Beta (95% Credible Interval)).*** *All models were adjusted for sub-cohort, maternal age, maternal pre-pregnancy BMI, maternal education level parity, child sex and child age.*

© 2022 Midya V et al. *JAMA Network Open*.

eTable 6. Estimated Posterior Weights of Exposure Mixture Groups on Liver Injury and CK-18 Outcomes Using BWQS Models

	Liver Injury Risk (95% CrI)	*CK-18 (95% CrI)*
DDE	0.57 (0.19, 0.88)	0.33 (0.01, 0.84)
DDT	0.29 (0.02, 0.65)	0.32 (0.01, 0.81)
HCB	0.14 (0.01, 0.38)	0.36 (0.020, 0.85)
PCB118	0.22 (0.01, 0.62)	0.32 (0.03, 0.67)
PCB138	0.20 (0.01, 0.60)	0.21 (0.01, 0.57)
PCB153	0.19 (0.01, 0.59)	0.15 (0.01, 0.46)
PCB170	0.20 (0.01, 0.60)	0.13 (0.00, 0.40)
PCB180	0.19 (0.01, 0.62)	0.20 (0.01, 0.57)
PBDE153	0.10 (0.01, 0.29)	0.84 (0.58, 0.99)
PBDE47	0.90 (0.71, 1.00)	0.16 (0.01, 0.42)
BPA	0.75 (0.51, 0.95)	0.27 (0.01, 0.74)
OXBE	0.18 (0.02, 0.41)	0.29 (0.02, 0.74)
TCS	0.07 (0.00, 0.22)	0.43 (0.03, 0.86)
BUPA	0.25 (0.01, 0.69)	0.25 (0.09, 0.70)
ETPA	0.23 (0.01, 0.67)	0.37 (0.02, 0.80)
MEPA	0.22 (0.01, 0.66)	0.18 (0.01, 0.57)
PRPA	0.31 (0.01, 0.74)	0.21 (0.01, 0.64)
MEHP	0.12 (0.00, 0.37)	0.14 (0.00, 0.4)
MEOHP	0.15 (0.01, 0.45)	0.14 (0.01, 0.46)
MEHHP	0.12 (0.00, 0.36)	0.13 (0.00, 0.42)
MECPP	0.14 (0.00, 0.41)	0.14 (0.00, 0.45)
MBzP	0.24 (0.01, 0.53)	0.16 (0.01, 0.50)
OHMiNP	0.10 (0.00, 0.31)	0.14 (0.01, 0.47)
OXOMiNP	0.13 (0.01, 0.37)	0.15 (0.00, 0.49)
MEP	0.35 (0.02, 0.81)	0.51 (0.02, 0.93)
MiBP	0.33 (0.02, 0.82)	0.24 (0.01, 0.74)
MnBP	0.32 (0.01, 0.84)	0.24 (0.00, 0.86)
DEP	0.25 (0.01, 0.69)	0.25 (0.01, 0.66)
DETP	0.25 (0.01, 0.74)	0.23 (0.01, 0.65)
DMP	0.25 (0.02, 0.67)	0.24 (0.01, 0.67)

© 2022 Midya V et al. *JAMA Network Open*.

DMTP	0.26 (0.02, 0.73)	0.29 (0.02, 0.75)
PFHxS	0.05 (0.00, 0.15)	0.21 (0.01, 0.61)
PFNA	0.43 (0.15, 0.73)	0.22 (0.01, 0.61)
PFOA	0.42 (0.13, 0.71)	0.22 (0.01, 0.61)
PFOS	0.05 (0.00, 0.16)	0.18 (0.01, 0.55)
PFUnDA	0.06 (0.00, 0.18)	0.17 (0.00, 0.55)
As	0.08 (0.00, 0.22)	0.09 (0.00, 0.29)
Cd	0.09 (0.01, 0.22)	0.10 (0.00, 0.31)
Co	0.13 (0.02, 0.28)	0.11 (0.00, 0.34)
Cs	0.07 (0.00, 0.20)	0.18 (0.01, 0.44)
Cu	0.06 (0.00, 0.17)	0.13 (0.01, 0.35)
Hg	0.40 (0.23, 0.58)	0.09 (0.00, 0.30)
Mn	0.03 (0.00, 0.10)	0.07 (0.00, 0.25)
Mo	0.08 (0.00, 0.21)	0.17 (0.01, 0.41)
Pb	0.05 (0.00, 0.15)	0.08 (0.00, 0.27)

Note: ***As***, Inorganic arsenic; ***BPA***, Bisphenol A; ***BUPA***, N-butyl paraben; ***Cd***, Cadmium; ***Cs***, Caesium; ***Co***, Cobalt; ***Cu,*** Copper; ***DDE***, 4,4′dichlorodiphenyl dichloroethylene; ***DDT***, 4,4′dichlorodiphenyltrichloroethane; ***DEP***, diethyl phosphare; ***DEHP***, di(2-ethylhexyl) phthalate; ***DETP***, diethyl thiophosphate; ***DEDTP***, diethyl dithiophosphate; ***DMP***, dimethyl phosphate; ***DMTP***, dimethyl thiophosphate; ***DMDTP***, dimethyl dithiophosphate; ***ETPA***, ethyl paraben; ***HCB***, hexachlorobenzene; ***Hg***, Mercury; ***MBzP***, mono benzyl phthalate; ***MECPP***, mono-2-ethyl 5-carboxypentyl phthalate; ***MEHP***, mono(2-ethylhexyl) phthalate; ***MEHHP***, mono(2-ethyl-5-hydroxyhexyl) phthalate; ***MEOHP***, mono(2-ethyl-5-oxohexyl) phthalate; ***MEP***, monoethyl phthalate; ***MEPA***, methyl paraben; ***MiBP***, mono-iso-butyl phthalate; ***Mn***, Manganese; ***MnBP***, mono-n-butyl phthalate; ***Mo***, Molybdenum; ***OHMiNP***, mono-4-methyl-7-hydroxyoctyl phthalate; ***OXBE***, oxybenzone; ***OXOMiNP***, mono-4-methyl-7-oxooctyl phthalate; ***Pb***, Lead; ***PBDE47***, 2,2′,4,4′-tetra-bromodiphenyl ether; ***PBDE153***, 2,2′,4,4′,5,5′-hexa-bromodiphenyl ether; ***PCBs***, polychlorinated biphenyl –118, 138, 153, 170, 180; ***PFHxS***, Perfluorohexane sulfonate; ***PFNA***, perfluorononanoate; ***PFOA***, perfluorooctanoate; ***PFOS***, perfluorooctane sulfonate; ***PFUnDA***, perfluoroundecanoate; ***PRPA,*** propyl paraben; ***TCS***, triclosan; ***CrI***, Credible Interval; *Estimated weights (contribution of each chemical to the overall group association) are based on the BWQS model for liver Injury Risk and CK-18. For each chemical group, the estimated weights sum up to one, therefore implying the relative contribution of each exposure to the overall group mixture association.*

© 2022 Midya V et al. *JAMA Network Open*.

eTable 7. BKMR Models for Liver Injury and CK-18

		Liver Injury Risk		CK-18	
	Quantile*	Beta	95% CI	Beta	95% CI
**OC Pesticides**	25th	-0.04	(-0.10, 0.02)	-0.17	(-2.59, 2.24)
	50th	0	0	0	0
	75th	0.07	(-0.00, 0.14)	-1.07	(-4.05, 1.91)
**PCBs**	25th	-0.01	(-0.07, 0.05)	-0.58	(-3.10, 1.93)
	50th	0	0	0	0
	75th	0.05	(-0.01, 0.12)	0.98	(-1.82, 3.79)
**PBDEs**	25th	-0.01	(-0.05, 0.04)	-4.35	(-7.51, -1.19)
	50th	0	0	0	0
	75th	0.03	(-0.03, 0.09)	3.58	(0.51, 6.64)
**Phenols**	25th	0.04	(-0.04, 0.13)	-5.74	(-9.85, -1.63)
	50th	0	0	0	0
	75th	-0.04	(-0.15, 0.08)	3.40	(-2.62, 9.42)
**Parabens**	25th	-0.01	(-0.12, 0.09)	0.5	(-1.81, 3.10)
	50th	0	0	0	0
	75th	-0.01	(-0.09, 0.07)	-0.18	(-2.51, 2.15)
**HMWPs**	25th	0.05	(-0.02, 0.11)	-0.01	(-0.54, 0.52)
	50th	0	0	0	0
	75th	-0.09	(-0.16, -0.02)	0.00	(-0.66, 0.67)
**LMWPs**	25th	-0.01	(-0.07, 0.04)	-0.65	(-4.16, 2.87)
	50th	0	0	0	0
	75th	0.02	(-0.05, 0.08)	-1.38	(-5.29, 2.52)
**OP Pesticides**	25th	0.02	(-0.07, 0.10)	-0.15	(-1.62, 1.32)
	50th	0	0	0	0
	75th	0.02	(-0.05, 0.09)	0.16	(-1.56, 1.88)
**PFAS**	25th	-0.08	(-0.14, -0.02)	0.11	(-1.99, 2.20)
	50th	0	0	0	0
	75th	0.08	(0.02, 0.15)	-0.30	(-2.51, 1.91)
**Metals**	25th	-0.08	(-0.21, 0.05)	-0.03	(-0.62, 0.56)
	50th	0	0	0	0
	75th	0.14	(0.03, 0.26)	0.01	(-0.35, 0.37)

**Difference in effect sizes (and corresponding 95% CIs) when all the exposures in a group were at a particular percentile (25th or 75th) with respect to when all were fixed at 50th percentile for Liver Injury risk and CK-18. All models were adjusted for sub-cohort, maternal age, maternal pre-pregnancy BMI, maternal education level parity, child sex and child age.*

© 2022 Midya V et al. *JAMA Network Open*.

eTable 8. Estimated Posterior Inclusion Probabilities (PIPs) of Exposures in the Mixture Groups for Liver Injury and CK-18 Outcomes Using BKMR Models

	Liver Injury Risk	CK-18
**DDE**	0.27	0.31
**DDT**	0.43	0.38
**HCB**	0.31	0.30
**PCB118**	0.18	0.24
**PCB138**	0.13	0.16
**PCB153**	0.25	0.34
**PCB170**	0.23	0.07
**PCB180**	0.21	0.21
**PBDE153**	0.38	0.88
**PBDE47**	0.62	0.12
**BPA**	0.55	0.13
**OXBE**	0.25	0.01
**TCS**	0.20	0.87
**BUPA**	0.20	0.24
**ETPA**	0.17	0.26
**MEPA**	0.20	0.25
**PRPA**	0.42	0.26
**MEHP**	0.13	0.16
**MEOHP**	0.16	0.15
**MEHHP**	0.22	0.12
**MECPP**	0.16	0.13
**MBzP**	0.09	0.13
**OHMiNP**	0.14	0.16
**OXOMiNP**	0.11	0.15
**MEP**	0.11	0.39
**MiBP**	0.41	0.31
**MnBP**	0.48	0.30
**DEP**	0.18	0.24
**DETP**	0.20	0.25
**DMP**	0.14	0.25

© 2022 Midya V et al. *JAMA Network Open*.

**DMTP**	0.48	0.26
**PFHxS**	0.13	0.21
**PFNA**	0.28	0.19
**PFOA**	0.26	0.21
**PFOS**	0.22	0.19
**PFUnDA**	0.11	0.21
As	0.06	0.11
Cd	0.06	0.12
Co	0.09	0.11
Cs	0.06	0.10
Cu	0.10	0.12
Hg	0.45	0.11
Mn	0.10	0.10
Mo	0.04	0.11
Pb	0.05	0.12

Note: ***As***, Inorganic arsenic; ***BPA***, Bisphenol A; ***BUPA***, N-butyl paraben; ***Cd***, Cadmium; ***Cs***, Caesium; ***Co***, Cobalt; ***Cu,*** Copper; ***DDE***, 4,4′dichlorodiphenyl dichloroethylene; ***DDT***, 4,4′dichlorodiphenyltrichloroethane; ***DEP***, diethyl phosphare; ***DEHP***, di(2-ethylhexyl) phthalate; ***DETP***, diethyl thiophosphate; ***DEDTP***, diethyl dithiophosphate; ***DMP***, dimethyl phosphate; ***DMTP***, dimethyl thiophosphate; ***DMDTP***, dimethyl dithiophosphate; ***ETPA***, ethyl paraben; ***HCB***, hexachlorobenzene; ***Hg***, Mercury; ***MBzP***, mono benzyl phthalate; ***MECPP***, mono-2-ethyl 5-carboxypentyl phthalate; ***MEHP***, mono(2-ethylhexyl) phthalate; ***MEHHP***, mono(2-ethyl-5-hydroxyhexyl) phthalate; ***MEOHP***, mono(2-ethyl-5-oxohexyl) phthalate; ***MEP***, monoethyl phthalate; ***MEPA***, methyl paraben; ***MiBP***, mono-iso-butyl phthalate; ***Mn***, Manganese; ***MnBP***, mono-n-butyl phthalate; ***Mo***, Molybdenum; ***OHMiNP***, mono-4-methyl-7-hydroxyoctyl phthalate; ***OXBE***, oxybenzone; ***OXOMiNP***, mono-4-methyl-7-oxooctyl phthalate; ***Pb***, Lead; ***PBDE47***, 2,2′,4,4′-tetra-bromodiphenyl ether; ***PBDE153***, 2,2′,4,4′,5,5′-hexa-bromodiphenyl ether; ***PCBs***, polychlorinated biphenyl –118, 138, 153, 170, 180; ***PFHxS***, Perfluorohexane sulfonate; ***PFNA***, perfluorononanoate; ***PFOA***, perfluorooctanoate; ***PFOS***, perfluorooctane sulfonate; ***PFUnDA***, perfluoroundecanoate; ***PRPA,*** propyl paraben; ***TCS***, triclosan;

For each chemical group, the estimated PIPs were scaled to sum up to one, therefore implying the relative inclusion of each chemical exposure to the overall group mixture association.

© 2022 Midya V et al. *JAMA Network Open*.

eTable 9. Linear Mixed Effects Regression (LMER) Models for Individual Chemicals on Binary Liver Injury and Continuous CK-18 Outcomes

	Liver Injury Risk				CK-18			
	**OR**	**95% CI**	**pvalue**	**qvalue**	**Beta**	**95% CI**	**pvalue**	**qvalue**
**DDE**	1.08	(0.91, 1.29)	0.37	0.58	-0.70	(-3.87, 2.47)	0.66	0.97
**DDT**	1.21	(1.04, 1.41)	0.01	0.27	-0.93	(-3.77, 1.91)	0.52	0.91
**HCB**	1.07	(0.88, 1.30)	0.50	0.64	0.11	(-3.25, 3.48)	0.95	0.99
**PCB118**	1.11	(0.90, 1.36)	0.33	0.56	2.11	(-1.40, 5.63)	0.24	0.91
**PCB138**	1.05	(0.85, 1.29)	0.68	0.75	1.60	(-1.87, 5.06)	0.37	0.91
**PCB153**	1.27	(0.78, 1.19)	0.74	0.79	1.29	(-2.39, 4.92)	0.50	0.91
**PCB170**	1.05	(0.86, 1.29)	0.63	0.74	-1.43	(-4.97, 2.12)	0.43	0.91
**PCB180**	0.89	(0.67, 1.05)	0.12	0.44	2.44	(-1.31, 6.19)	0.20	0.91
**PBDE153**	1.06	(0.91, 1.24)	0.48	0.64	5.88	(3.03, 8.74)	0.00	0.00
**PBDE47**	1.13	(0.95, 1.35)	0.16	0.53	0.42	(-2.59, 3.43)	0.78	0.99
**BPA**	0.85	(0.72, 1.00)	0.04	0.33	-0.16	(-3.12, 2.81)	0.92	0.99
**OXBE**	0.92	(0.80, 1.07)	0.29	0.55	0.94	(-1.75, 3.63)	0.49	0.91
**TCS**	1.06	(0.91, 1.23)	0.47	0.64	2.74	(0.05, 5.43)	0.05	0.62
**BUPA**	1.05	(0.90, 1.21)	0.57	0.69	-1.80	(-4.62, 1.02)	0.21	0.91
**ETPA**	0.99	(0.85, 1.15)	0.88	0.79	-2.27	(-5.01, 0.47)	0.10	0.79
**MEPA**	0.99	(0.85, 1.14)	0.84	0.79	0.49	(-2.13, 3.11)	0.71	0.97
**PRPA**	0.99	(0.85, 1.14)	0.87	0.79	-1.35	(-4.03, 1.34)	0.32	0.91
**MEHP**	0.91	(0.79, 1.06)	0.21	0.53	-0.87	(-3.68, 1.94)	0.54	0.91
**MEOHP**	0.84	(0.73, 0.98)	0.02	0.27	0.82	(-1.83, 3.48)	0.54	0.91
**MEHHP**	0.84	(0.73, 0.97)	0.02	0.27	0.09	(-2.58, 2.75)	0.95	0.99
**MECPP**	0.87	(0.76, 1.00)	0.05	0.34	-0.32	(-2.90, 2.25)	0.81	0.99
**MBzP**	0.91	(0.77, 1.08)	0.28	0.55	1.07	(-1.82, 3.97)	0.47	0.91
**OHMiNP**	0.99	(0.85, 1.16)	0.93	0.79	-1.35	(-4.14, 1.44)	0.34	0.91
**OXOMiNP**	0.86	(0.74, 1.00)	0.04	0.33	-0.02	(-2.67, 2.62)	0.99	0.99
**MEP**	1.03	(0.89, 1.20)	0.65	0.75	-2.62	(-5.26, 0.01)	0.05	0.62
**MiBP**	1.01	(0.87, 1.17)	0.92	0.79	-0.18	(-2.79, 2.44)	0.90	0.99
**MnBP**	1.00	(0.86, 1.15)	0.96	0.80	1.25	(-1.39, 3.89)	0.35	0.91
**DEP**	0.99	(0.86, 1.14)	0.90	0.79	0.53	(-2.04, 3.09)	0.69	0.97
**DETP**	1.08	(0.94, 1.25)	0.27	0.55	0.22	(-2.37, 2.80)	0.87	0.99

© 2022 Midya V et al. *JAMA Network Open*.

**DMP**	0.94	(0.81, 1.10)	0.46	0.64	0.89	(-1.79, 3.57)	0.52	0.91
**DMTP**	0.93	(0.81, 1.08)	0.35	0.57	1.63	(-0.98, 4.23)	0.22	0.91
**PFHxS**	1.12	(0.94, 1.34)	0.21	0.53	-1.40	(-4.67, 1.87)	0.40	0.91
**PFNA**	1.17	(0.97, 1.40)	0.11	0.44	-1.13	(-4.38, 2.13)	0.50	0.91
**PFOA**	1.16	(0.97, 1.38)	0.10	0.44	0.06	(-3.09, 3.20)	0.97	0.99
**PFOS**	1.10	(0.91, 1.33)	0.31	0.56	-0.64	(-3.98, 2.70)	0.71	0.97
**PFUnDA**	1.11	(0.94, 1.30)	0.23	0.53	0.49	(-2.41, 3.38)	0.74	0.98
As	1.10	(0.95, 1.28)	0.20	0.53	1.23	(-1.59, 4.04)	0.39	0.91
Cd	0.95	(0.82, 1.11)	0.52	0.66	0.01	(-2.69, 2.72)	0.99	0.99
Co	1.02	(0.88, 1.18)	0.80	0.79	-0.68	(-3.40, 2.03)	0.62	0.97
Cs	1.12	(0.94, 1.32)	0.20	0.53	-3.15	(-6.37, 0.070)	0.06	0.62
Cu	0.92	(0.80, 1.06)	0.26	0.55	-1.07	(-3.74, 1.60)	0.43	0.91
Hg	1.23	(0.96, 1.58)	0.10	0.44	2.99	(-0.27, 6.25)	0.07	0.65
Mn	0.99	(0.85, 1.14)	0.86	0.79	-0.33	(-3.08, 2.43)	0.82	0.99
Mo	0.94	(0.82, 1.09)	0.42	0.63	-1.66	(-4.27, 0.95)	0.21	0.91
Pb	0.99	(0.86, 1.14)	0.87	0.79	-0.56	(-3.19, 2.08)	0.68	0.97

Note: ***As***, Inorganic arsenic; ***BPA***, Bisphenol A; ***BUPA***, N-butyl paraben; ***Cd***, Cadmium; ***Cs***, Caesium; ***Co***, Cobalt; ***Cu,*** Copper; ***DDE***, 4,4′dichlorodiphenyl dichloroethylene; ***DDT***, 4,4′dichlorodiphenyltrichloroethane; ***DEP***, diethyl phosphare; ***DEHP***, di(2-ethylhexyl) phthalate; ***DETP***, diethyl thiophosphate; ***DEDTP***, diethyl dithiophosphate; ***DMP***, dimethyl phosphate; ***DMTP***, dimethyl thiophosphate; ***DMDTP***, dimethyl dithiophosphate; ***ETPA***, ethyl paraben; ***HCB***, hexachlorobenzene; ***Hg***, Mercury; ***MBzP***, mono benzyl phthalate; ***MECPP***, mono-2-ethyl 5-carboxypentyl phthalate; ***MEHP***, mono(2-ethylhexyl) phthalate; ***MEHHP***, mono(2-ethyl-5-hydroxyhexyl) phthalate; ***MEOHP***, mono(2-ethyl-5-oxohexyl) phthalate; ***MEP***, monoethyl phthalate; ***MEPA***, methyl paraben; ***MiBP***, mono-iso-butyl phthalate; ***Mn***, Manganese; ***MnBP***, mono-n-butyl phthalate; ***Mo***, Molybdenum; ***OHMiNP***, mono-4-methyl-7-hydroxyoctyl phthalate; ***OXBE***, oxybenzone; ***OXOMiNP***, mono-4-methyl-7-oxooctyl phthalate; ***Pb***, Lead; ***PBDE47***, 2,2′,4,4′-tetra-bromodiphenyl ether; ***PBDE153***, 2,2′,4,4′,5,5′-hexa-bromodiphenyl ether; ***PCBs***, polychlorinated biphenyl –118, 138, 153, 170, 180; ***PFHxS***, Perfluorohexane sulfonate; ***PFNA***, perfluorononanoate; ***PFOA***, perfluorooctanoate; ***PFOS***, perfluorooctane sulfonate; ***PFUnDA***, perfluoroundecanoate; ***PRPA,*** propyl paraben; ***TCS***, triclosan; *The estimates are presented as* ***(OR (95% Confidence Interval) and Beta (95% Confidence Interval)).*** *All models were adjusted for sub-cohort, maternal age, maternal pre-pregnancy BMI, maternal education level parity, child sex and child age.*

© 2022 Midya V et al. *JAMA Network Open*.

eTable 10. Analyses of Individual Liver Enzymes (ALT, AST, and GGT) Using BWQS Models

	ALT		GGT		AST	
	**Beta**	**95% CrI**	**Beta**	**95% CrI**	**Beta**	**95% CrI**
OC Pesticides	0.78	(0.29, 1.27)	0.40	(0.01, 0.72)	0.56	(-0.39, 1.17)
PCBs	0.12	(-0.48, 0.74)	-0.11	(-0.56, 0.34)	-0.03	(-0.82, 0.74)
PBDEs	1.09	(0.65, 1.53)	0.42	(-0.01, 0.73)	1.42	(0.93, 1.91)
Phenols	-0.95	(-1.37, -0.50)	-0.54	(-0.90, -0.18)	-1.08	(-1.68, -0.50)
Parabens	-0.15	(-0.66, 0.42)	-0.12	(-0.51, 0.29)	-0.35	(-0.97, 0.26)
HMWPs	-0.10	(-0.64, 0.44)	-0.25	(-0.64, 0.16)	-1.12	(-1.88, -0.34)
LMWPs	0.20	(-0.30, 0.72)	-0.05	(-0.40, 0.32)	-0.30	(-0.93, 0.35)
OP Pesticides	-0.03	(-0.50, 0.46)	-0.25	(-0.62, 0.12)	-0.14	(-0.77, 0.47)
PFAS	1.08	(0.63, 1.52)	0.83	(0.45, 1.19)	1.96	(1.38, 2.54)
Metals	2.61	(1.91, 3.27)	1.27	(0.67, 1.87)	2.80	(1.72, 3.87)

The estimates are presented as ***(Beta (95% Credible Interval)).*** *All models were adjusted for sub-cohort, maternal age, maternal pre-pregnancy BMI, maternal education level parity, child sex and child age.*

© 2022 Midya V et al. *JAMA Network Open*.

eTable 11. Stratified BWQS Analyses of Liver Injury and CK-18 by Sex of the Children

	Liver Injury Risk				CK-18			
	Male		Female		Male		Female	
	OR	95% CrI	OR	95% CrI	Beta	95% CrI	Beta	95% CrI
OC Pesticides	1.78	(1.40, 2.28)	1.13	(0.88, 1.48)	-0.24	(-4.35, 3.74)	-0.89	(-7.17, 4.89)
PCBs	1.03	(0.68, 1.57)	1.11	(0.75, 1.66)	3.11	(-1.14, 7.59)	7.93	(1.02, 14.72)
PBDEs	1.53	(1.23, 1.94)	1.59	(1.25, 2.05)	2.35	(-4.40, 6.81)	5.57	(-0.88, 11.16)
Phenols	0.65	(0.50, 0.85)	0.65	(0.48, 0.87)	0.70	(-3.40, 5.08)	5.32	(-2.13, 12.39)
Parabens	0.90	(0.71, 1.13)	0.97	(0.74, 1.25)	1.24	(-2.81, 5.42)	-7.35	(-13.60, -1.07)
HMWPs	0.87	(0.65, 1.16)	0.63	(0.45, 0.87)	0.63	(-3.88, 4.77)	-0.14	(-8.15, 8.13)
LMWPs	1.08	(0.85, 1.40)	0.87	(0.63, 1.26)	0.68	(-3.87, 4.96)	-3.49	(-10.77, 5.59)
OP Pesticides	0.90	(0.71, 1.16)	1.07	(0.81, 1.40)	1.87	(-2.09, 5.72)	1.12	(-4.95, 7.35)
PFAS	1.77	(1.37, 2.28)	1.68	(1.25, 2.27)	-0.48	(-5.22, 3.99)	-4.26	(-11.10, 2.55)
Metals	2.96	(1.95, 4.65)	1.54	(0.83, 2.62)	-3.28	(-10.86, 4.68)	-7.67	(-19.30, 3.15)

*The estimates are presented as **(OR (95% Credible Interval) or Beta (95% Credible Interval)).** All models were adjusted for sub-cohort, maternal age, maternal pre-pregnancy BMI, maternal education level parity, child sex and child age.*

© 2022 Midya V et al. *JAMA Network Open*.

eTable 12. BWQS Analyses of Liver Injury and CK-18 After Adjusting for Postnatal Chemical Exposures

	**Liver Injury Risk**		**CK-18**	
	**OR**	**95% CrI**	**Beta**	**95% CrI**
OC Pesticides	1.52	(1.22, 1.91)	-0.16	(-3.99, 3.72)
PCBs	1.07	(0.79, 1.45)	4.18	(-0.16, 8.61)
PBDEs	1.51	(1.27, 1.81)	5.46	(2.28, 8.51)
Phenols	0.68	(0.56, 0.83)	3.47	(-0.82, 7.52)
Parabens	0.95	(0.78, 1.14)	-2.01	(-5.65, 1.61)
HMWPs	0.75	(0.60, 0.92)	0.43	(-3.74, 4.66)
LMWPs	1.02	(0.83, 1.25)	-1.75	(-6.08, 3.01)
OP Pesticides	0.97	(0.80, 1.18)	1.59	(-1.88, 5.09)
PFAS	1.58	(1.29, 1.92)	-1.24	(-5.55, 2.81)
Metals	1.91	(1.36, 2.63)	-4.99	(-11.85, 1.97)

The estimates are presented as ***(OR (95% Credible Interval) or Beta (95% Credible Interval)).*** *All models were adjusted for sub-cohort, maternal age, maternal pre-pregnancy BMI, maternal education level parity, child sex and child age.*

© 2022 Midya V et al. *JAMA Network Open*.

eTable 13. BWQS Analyses of Liver Injury and CK-18 After Adjusting for Maternal Diet Quality (Consumption of Fish, Fruits, and Vegetables in Times per Week)

	Liver Injury Risk		CK-18	
	**OR**	**95% CrI**	**Beta**	**95% CrI**
OC Pesticides	1.40	(1.16, 1.68)	-0.95	(-4.66, 2.55)
PCBs	1.05	(0.78, 1.41)	5.64	(1.38, 9.93)
PBDEs	1.55	(1.32, 1.83)	4.89	(1.84, 7.79)
Phenols	0.68	(0.56, 0.82)	3.23	(-0.81, 7.35)
Parabens	0.94	(0.79, 1.12)	-2.03	(-5.72, 1.99)
HMWPs	0.72	(0.58, 0.89)	0.19	(-4.08, 4.40)
LMWPs	1.00	(0.81, 1.23)	-1.71	(-6.34, 3.43)
OP Pesticides	0.97	(0.81, 1.19)	1.34	(-2.24, 4.81)
PFAS	1.70	(1.41, 2.08)	-3.36	(-6.84, 0.27)
Metals	2.25	(1.63, 3.07)	-6.08	(-13.06, 0.68)

The estimates are presented as ***(OR (95% Credible Interval) or Beta (95% Credible Interval)).*** *All models were adjusted for sub-cohort, maternal age, maternal pre-pregnancy BMI, maternal education level parity, child sex and child age.*

© 2022 Midya V et al. *JAMA Network Open*.

eTable 14. BWQS Analyses of Liver Injury and CK-18 After Adjusting for Child BMI *z* Score

	Liver Injury Risk		CK-18	
	**OR**	**95% CrI**	**Beta**	**95% CrI**
OC Pesticides	1.42	(1.19, 1.70)	-0.54	(-4.32, 2.75)
PCBs	1.06	(0.79, 1.42)	5.99	(1.73, 10.13)
PBDEs	1.54	(1.31, 1.81)	6.47	(3.15, 9.91)
Phenols	0.66	(0.55, 0.79)	2.64	(-1.38, 6.72)
Parabens	0.91	(0.76, 1.08)	-2.59	(-6.04, 0.91)
HMWPs	0.74	(0.60, 0.92)	0.08	(-4.46, 4.54)
LMWPs	1.01	(0.83, 1.22)	-1.95	(-6.31, 3.57)
OP Pesticides	0.97	(0.81, 1.17)	1.78	(-1.43, 5.23)
PFAS	1.73	(1.43, 2.10)	-1.99	(-5.92, 1.84)
Metals	2.12	(1.58, 2.92)	-6.36	(-12.90, 0.74)

The estimates are presented as ***(OR (95% Credible Interval) or Beta (95% Credible Interval)).*** *All models were adjusted for sub-cohort, maternal age, maternal pre-pregnancy BMI, maternal education level parity, child sex and child age.*

© 2022 Midya V et al. *JAMA Network Open*.

eReferences

1. Maitre L, de Bont J, Casas M, et al. Human Early Life Exposome (HELIX) study: a European population-based exposome cohort. *BMJ Open*. Sep 10 2018;8(9):e021311. doi:10.1136/bmjopen-2017-021311
2. Vos MB, Barve S, Joshi-Barve S, Carew JD, Whitington PF, McClain CJ. Cytokeratin 18, a marker of cell death, is increased in children with suspected nonalcoholic fatty liver disease. *J Pediatr Gastroenterol Nutr*. Oct 2008;47(4):481-5. doi:10.1097/MPG.0b013e31817e2bfb
3. Colicino E, Pedretti NF, Busgang SA, Gennings C. Per- and poly-fluoroalkyl substances and bone mineral density: Results from the Bayesian weighted quantile sum regression. *Environ Epidemiol*. Jun 2020;4(3):e092. doi:10.1097/EE9.0000000000000092
4. Bobb JF, Valeri L, Claus Henn B, et al. Bayesian kernel machine regression for estimating the health effects of multi-pollutant mixtures. *Biostatistics*. Jul 2015;16(3):493-508. doi:10.1093/biostatistics/kxu058
5. Jedynak P, Maitre L, Guxens M, et al. Prenatal exposure to a wide range of environmental chemicals and child behaviour between 3 and 7 years of age - An exposome-based approach in 5 European cohorts. *Sci Total Environ*. Apr 1 2021;763:144115. doi:10.1016/j.scitotenv.2020.144115
6. Haug LS, Sakhi AK, Cequier E, et al. In-utero and childhood chemical exposome in six European mother-child cohorts. *Environ Int*. Dec 2018;121(Pt 1):751-763. doi:10.1016/j.envint.2018.09.056
7. Vrijheid M, Fossati S, Maitre L, et al. Early-Life Environmental Exposures and Childhood Obesity: An Exposome-Wide Approach. *Environ Health Perspect*. Jun 2020;128(6):67009. doi:10.1289/EHP5975

© 2022 Midya V et al. *JAMA Network Open*.
